# Exploring traditional mongolian materia medica: the path of progress from tradition to modernity

**DOI:** 10.3389/fphar.2025.1554448

**Published:** 2025-07-29

**Authors:** Ruyu Shi, Enironggui Wang, Mengyang Wang, Chunhong Zhang, Tsend-Ayush Damda, Minhui Li

**Affiliations:** ^1^ Institute of Chinese Materia Medica, Inner Mongolia Autonomous Region Hospital of Traditional Chinese Medicine, Hohhot, China; ^2^ College of Pharmacy, Inner Mongolia Minzu University, Tongliao, China; ^3^ College of Pharmacy, Mongolian National University of Medical Sciences (MNUMS), Ulaanbaatar, Mongolia

**Keywords:** mongolian materia medica, resources, chemical composition, pharmacology, challenges

## Abstract

Traditional Mongolian medicine boasts a long history with its integrated and unigue theoretical system. Traditional Mongolian materia medica (TMMM), as the primary substance for the treatments in traditional Mongolian medicine, was thought to be an important source for drug discovery. Although interest in TMMM has recently grown among researchers, challenges of TMMM revitalisation should be concerned: (1) inheritors and sustainability, (2) effective composition and pharmacology, and (3) preparation and standardization. The purpose of the review is to introduce the current development of Mongolian medicine and summarize the future challenges to make better use of Mongolian medicine.

## 1 Introduction

For centuries, through the process of hunting for food or edible substances, humans have discovered materials with medicinal properties (Li et al., 2015). The curtain rose on traditional medicine applications millennia ago. People with specific cultural beliefs and habitats have developed different medical systems worldwide, using the available natural botanical drugs and mineral resources. Traditional medicines include Ayurveda, traditional Chinese medicine (TCM), Siddha medicine, Unani, ancient Iranian medicine, Islamic medicine, Irani, traditional Korean medicine and traditional African medicine (Wang, 2003). The Mongolian lifestyle and culture have also influence traditional medicine. Historically, Mongolians have mainly been dispersed across the Eurasian grassland; however, since the time of Genghis Khan, their scope of activity has expanded (Wang, 2003). Around the seventh century, Mongolian tribes were strengthening. Traditional Mongolian medicine gradually improved along with the tribes (Natsagdorj, 2011; Tang, 2009). In terms of habits and customs, nomadism is the traditional way of life for Mongolian nationality. They consume high quantities of meat and dairy products (Wang, 2003; Pitschmann, et al., 2013), and thus created a medical method well-suited to their natural environment and living customs (Pitschmann et al., 2013).

In terms of culture, Mongolian medicine developed its own traditions and perspectives and was taught in independent medical schools in the thirteen century (Shagdarsuren, 1989). Around the 16th century, Tibetan medicine was introduced into Mongolia, and Lamaism became the main religion of the Mongolian people, which greatly affected the daily life of Mongolians, their religious practice, and the education of Mongolian physicians (Kletter, C et al., 2008). In the 17th century, the Tibetan language was introduced as a medical language, whereafter, Tibetan recipes were gradually translated into Mongolian (Gerke and Barbara, 2004). Today, the Mongolian Medical Heritage is officially recognized as a part of traditional medicine (Gerke and Barbara, 2004; Unkrig, 2002).

In ancient times, traditional Mongolian medicine absorbed the theories of Indian and Tibetan medicine, such as the five elements, Heyi, Xila, and Badagan, seven essential substances, three wastes, zang-fu organs, and white pulse theory, which laid the foundation for the systematization of Mongolian medical theory (Tuosigen. 2022). After the introduction of Indian and Tibetan medicine into the Mongolian region, Mongolian medical circles increasingly studied *Yijing Bazhi* and *Sibu Yidian*, combining them with traditional Mongolian medical theories and clinical experience. This led to the emergence of a large number of *Xionggen Eumuqi* (classical physicians) and the publication of nearly 100 Mongolian medical classics, including the representative works: *Mijue Fanghai* (Jambalchoidan, 2020), *Mengyao Zhengdian*, *Ganlu Sibu* (Liu, 2006). Mongolian medicine researchers have combined traditional Mongolian medicine with the fundamental theories of Han Chinese medicine, Tibetan medicine and Indian medicine, and have compiled a large number of classic works on Mongolian medicine. For instance, the Yuan Dynasty doctor Sadamishi studied and applied the acupuncture techniques of Han medicine and wrote the book *Ruizhu Tang Jingyan Fang* (Ai, 2020) which records many precious prescriptions.

**FIGURE 1:**
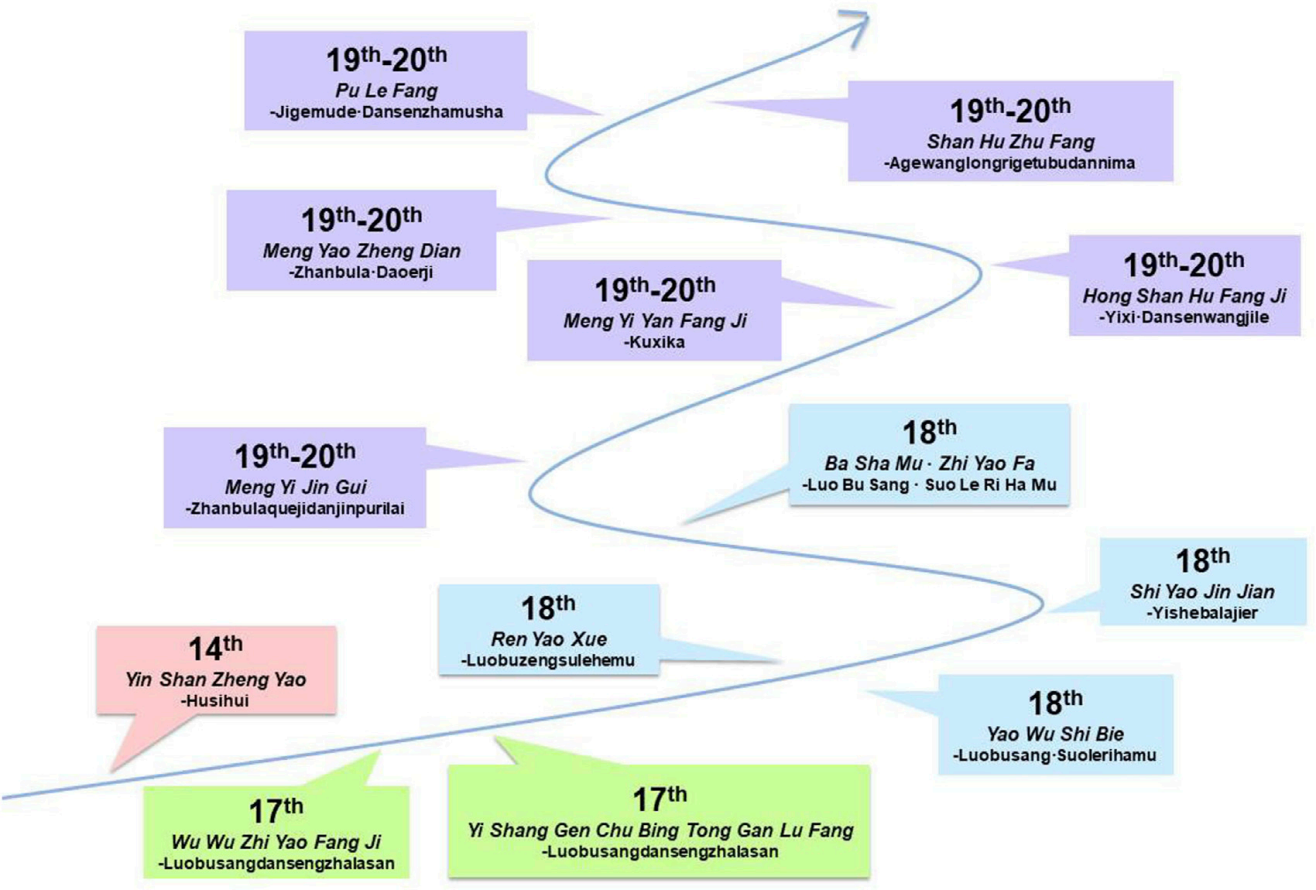
The time axis of representative TMMM works of A.D. 14th ∼20th. In chronological order: A.D.-14th Yin Shan Zheng Yao; A.D.-17th Wu Wu Zhi Yao Fang Ji; A.D.-18th Ren Yao Xue; A.D.-18th Yao Wu Shi Bie; A.D.-19th ∼20th Meng Yao Zheng Dian; A.D.-19th ∼20th Hong Shan Hu Fang Ji; A.D.-17th Yi Shang Gen Chu Bing Tong Gan Lu Fang; A.D.-18th Gan Lu Si Bu; A.D.-18th Shi Yao Jin Jian; A.D.-19th ∼20th Meng Yi Jin Gui; A.D.-19th ∼20th Meng Yi Yan Fang Ji; A.D.-19th ∼20th Pu Le Fang; A.D.-19th ∼20th Shan Hu Zhu Fang.

Numerous classic works have been produced in the long history of Mongolian medicine, which was in its infancy prior to the 12th century. Following the formation of the Mongolian pharmacy during the 13th-16th centuries (Robesan, 1922) and promoted by economic and cultural development, it was not until the 13th century that Hu Sihui of the Yuan Dynasty compiled a book on nutrition called Yin Shan Zheng Yao, which collected the prescription knowledge of famous doctors in previous dynasties and recorded the dietary principles and therapeutic formulae of the Mongolian people (Buren, 2007; Niu et al., 2024). After the 16th century, traditional Mongolian medicine underwent a period of rapid development (Zhou and Guan, 2007) during which many representative Mongolian medical scientists became prominent, compiling dozens of classic medical works. In the 18th century AD, the renowned Mongolian medical scientist Ihibalazur proposed the theory of the “Six Basic Diseases” (Heyi, Xila, Badagan, Qisu, Xiri Wusu, Nian) with distinct Mongolian medical characteristics in his work *Ganlu Huiji*. Some classic works of Tibetan medicine have been disseminated to the Mongolian region, including the ancient Indian medical masterpiece *Asurveda*, the ancient Indian Tripitaka *Kangyur*, *Danjur*, *Jinlan Xunjing*, *Sibu Yidian*, *Ren Yao Xue* and other medical books (Tuosigen, 2022). These books have been translated into Mongolian and disseminated. It has played an important role in promoting the development of Mongolian medicine. Mongolian pharmacists continued to summarize and expand the theory and practice of medicine, leading to the development of the major founding works of the TMMM (Figure 1), including the *Ren Yao Xue* (Luobuzengsulehemu, A.D.-18th), *Meng Yao Zheng Dian* (ZhanbulaDaoerji, A.D.-19th∼20th), and *Meng Yi Jin Gui* (Li et al., 2023) (Zhanbulaquejidanjinpurilai, A.D.-19th∼20th). The development of TMMM entered a new stage in the 1950s. Since the founding of the People’s Republic of China, the state has attached great importance to and encouraged the development of Mongolian medicine, organizing the compilation of more than a dozen ancient books on Mongolian treatment and prescriptions. These ancient books have become precious documents and are of immense significance for studying of Mongolian medical herbology.

The basic theory of Mongolian medicine is guided by the holistic concept and uses syndrome differentiation and treatment as its practical method (Suriya, 2012). It emphasizes the harmonious unity of the human body and nature. The theory of the three elements is one of the core contents of the basic theory of Mongolian medicine. The so-called three elements refer to Heyi, Xila and Badagan. These three are the main material bases that constitute the human body. Due to the physiological activities of the Heyi, Xila, and Badagan themselves, as well as the influence of people’s diet, daily life, seasons, and other factors, the Heyi, Xila, and Badagan have the possibility of excessive growth or depletion, leading to a loss of coordination. Therefore, the three elements have become one of the internal causes of diseases in people. Whenever any one or more of the three factors in the trinity show excessive proliferation or attenuation, they become pathological substances, namely, the three evils, presenting as Heyi pathology, Xila pathology or Badagan pathology. In terms of treatment, it is necessary to adjust the three to restore their original relative balanced state, so that life activities can proceed normally. Therefore, the three are interdependent, mutually restrictive, closely related, and coordinated and unified, thereby maintaining the operation and stability of the internal environment of the human body (Wang et al., 2018).

To date, in the era of massive information accessibility and artificial intelligence, after gradually unifying the chemical data characteristics of TMMM, we can compare the data of different research institutions and different periods. Therefore, analysis based on the comprehensive quantitative comparative chemical metabolites of TMMM is a comprehensive quality control system that identifies varieties of distinct elements, controls their quality, and limits harmful metabolites, which is more in line with the characteristics of TMMM. The application of TMMM chemical holography in the field of TMMM is a beneficial explorative exercise to promote the reform of TMMM quality control in the era of massive data availability. In general, contemporary studies in analytical chemistry allow for further expansion of TMMM. At the same time, resources, active metabolites, pharmacology, sustainability, and standardisation constitute the main framework of TMMM research, and the role of each part cannot be ignored. Compared to other traditional ethnic medicines, Mongolian medicine remains relatively underdeveloped. Current research reveals several critical limitations: insufficient investigation into the material basis of Mongolian drugs, a lack of systematic pharmacological studies, unclear pharmacokinetic properties, ambiguous compatibility principles in formulation, and slow progress in the development of new Mongolian medicines (Song et al., 2021). Furthermore, the decrease in existing TMMM resources has placed tremendous pressure on the development of Mongolian medicine, making it necessary to protect the native sources of these medicines, produce and utilize them efficiently, and increase awareness of the need to protect them and ensure their sustainability. This study aims to systematically evaluate key challenges in TMMM by analyzing its historical origins, current clinical applications, and therapeutic practices, as well as identifying areas requiring further clinical research. We synthesize evidence and propose recommendations for establishing standardized efficacy evaluation systems and advancing mechanistic studies of TMMM. Specifically, we conduct a comprehensive analysis of the chemical metabolites and pharmacological properties of Mongolian medicinal metabolites. Our focus encompasses three critical dimensions: (1) the empirical foundations of traditional practices, (2) scientific advancements in TMMM research, and (3) persistent challenges in resource utilization, phytochemistry, pharmacology, formulation, and dosage form development.

## 2 Methodology and literature search strategy

In this systematic review, a literature search was conducted on various sources, including Embase-Elsevier, PubMed, Google Scholar, Baidu Scholar, China National Knowledge Infrastructure, and Web of Science. A combination of the following keywords was used: Mongolian materia medica, Mongolian medicine, Mongolian medicinal plant, history, resources, active metabolites, pharmacology, bioactivity, sustainability, standardisation. This systematic review identified studies published from 2001 to 2024 to provide data on TMMM. Additional works related to Mongolian history were identified through the literature. With the keyword “Mongolian materia medica” as the core and with “resources, active metabolites, standardisation” as the limit, we retrieved many documents. The following criteria were then applied: (1) the most cited academic papers in the past 24 years, containing internationally acclaimed research, were collected; (2) duplicated publications, case reports, and examinations were removed; (3) simple *in vitro* studies without positive controls were also excluded from the pharmacological research. After removing duplicates and items that did not meet the criteria and selecting the most recent references from each retrieval, 184 studies and two books were included and divided into the following categories: i) The ‘resources’ category, which mainly focused on the collection and discovery of TMMM and the latest technical progress related to the protection of TMMM resources; ii) The ‘pharmacology’ category, which focused on the mechanisms underlying the effects of the latest active metabolites from TMMM, with *in vivo* experiments as the priority. For studies of complex dosage forms, we focused on frequently used and effective preparations. We examined studies focusing on the quality of raw materials and traditional Chinese medicines, as well as those describing challenges in the international standardisation of TMMM.

**FIGURE 2:**
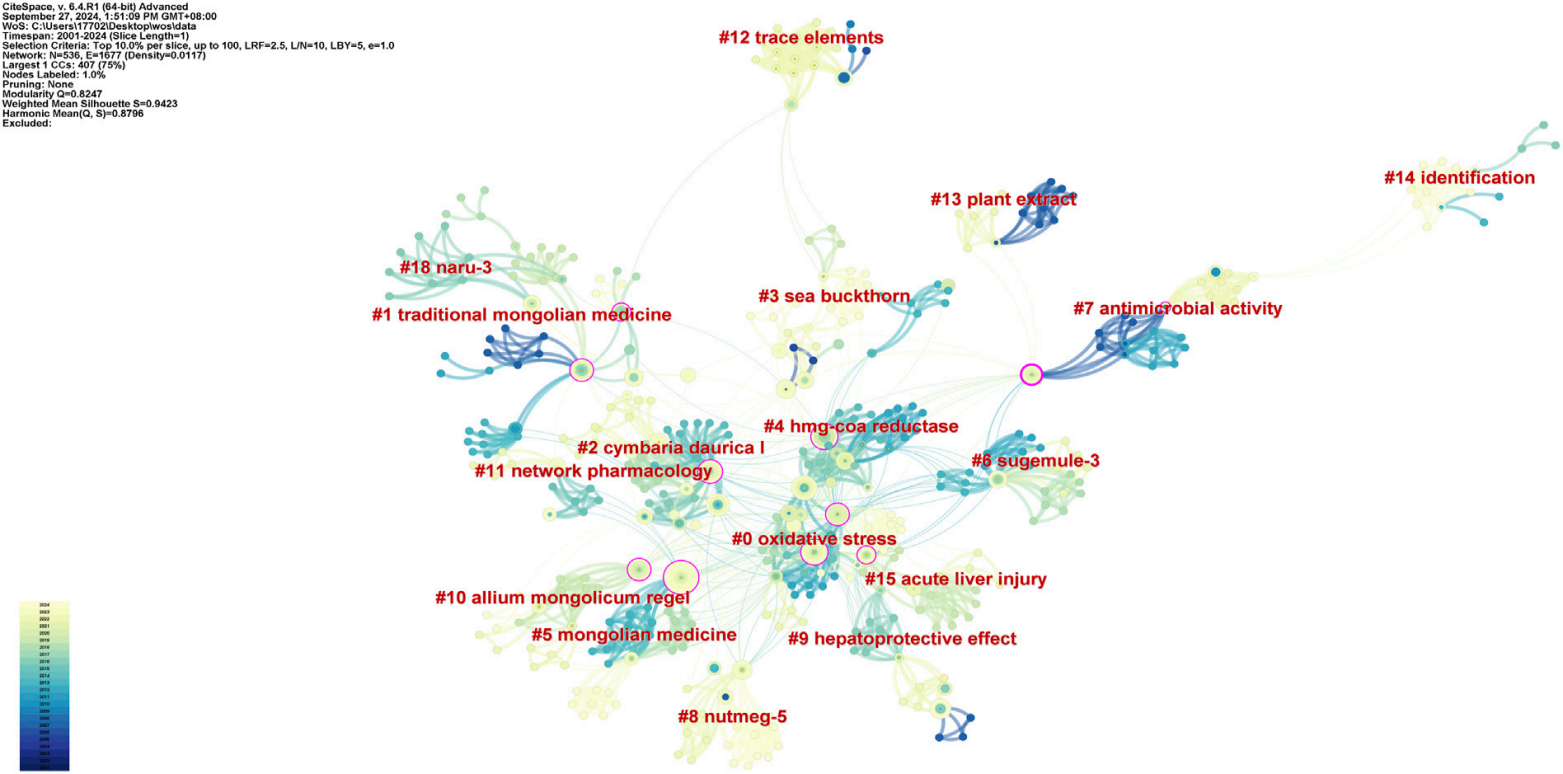
Keywords cluster co-occurrence atlas. The node size represents the strength of key-words; the links represent the relationship of these keywords.

**FIGURE 3:**
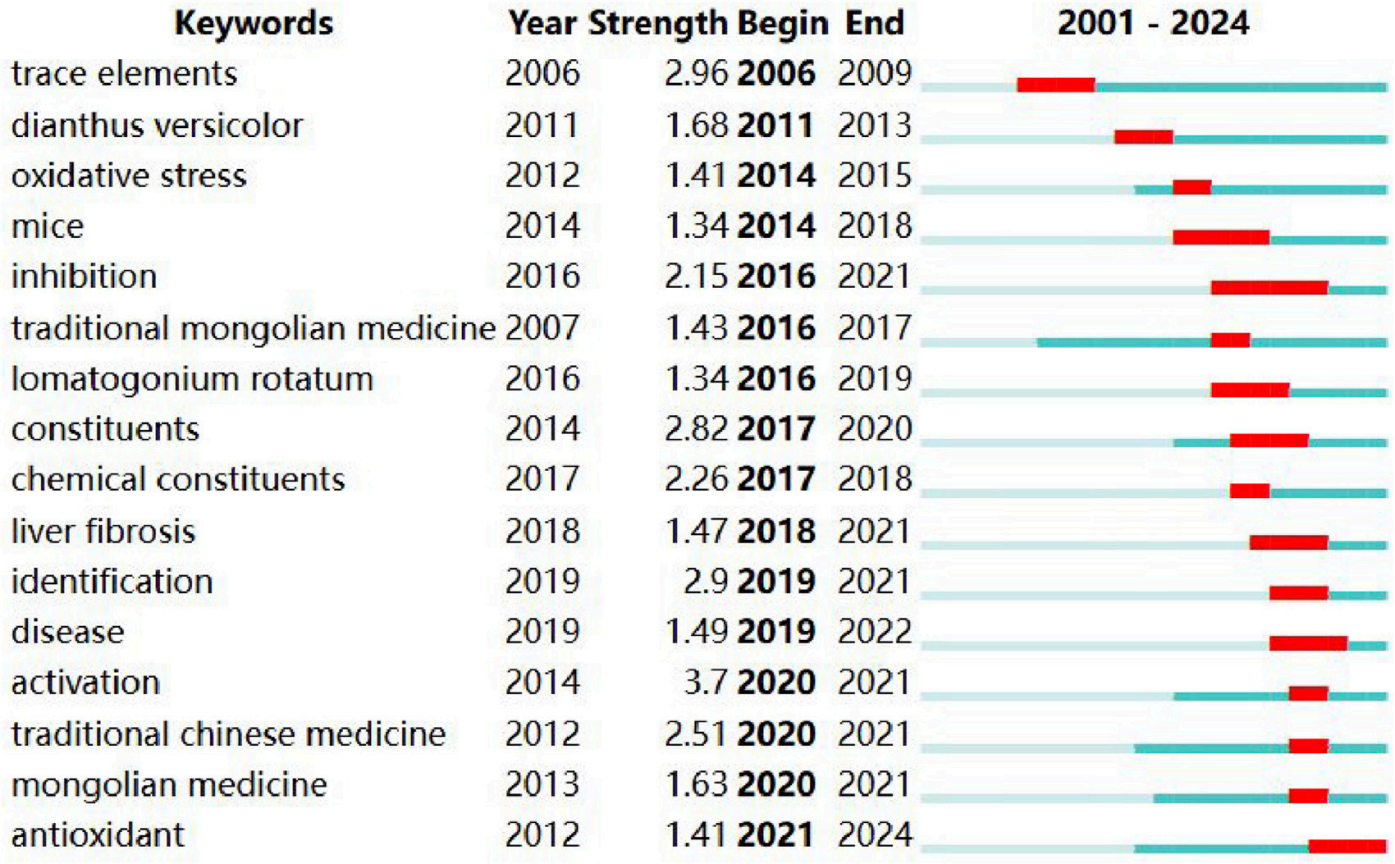
Top 16 keywords with the Strongest Citation Bursts. γ = [0.4]. Minimum duration = 1.

We conducted keyword clustering analysis and Frontier hot spot analysis on literature published in the last 24 years. In CiteSpace software, the time span was set to 2001–2024, time slicing was set 1, the node type selected was keyword, and Top% N was set as 100%. The parameter threshold was set as 0. The software was set to cluster by keyword. According to modularity Q = 0.8247, silhouette S = 0.9423; therefore, the data are reliable (Supplementary Figure 2). The cluster analysis showed that the seven keywords used with the highest frequency were oxidative stress, traditional mongolian medicine,3-Hydroxy-3-Methylglutaryl-Coenzyme A Reductase (HMG-CoA Reductase), antimicrobial activity, hepatoprotective effect, network pharmacology, acute liver injury. These keywords authentically reflect the current situation in traditional medicine research, which focuses on the pharmacological mechanisms and chemical metabolites exerting effects according to traditional applications. Specifically, apoptosis and the hepatoprotective effect of Mongolian medicine is a research hotspot in the pharmacological mechanism of Mongolian medicine at present. Related studies are also concerned with the health benefits of Mongolian medicine (Figure 2). The first occurrence and development of the keywords in each cluster are shown in Supplementary Figure 2. The keyword “oxidative stress” appeared first, which reflects the fact that research began to pay attention to the interaction between Mongolian medicine and cells in the early stages. This was also the first step taken to ascertain the mechanisms of Mongolian medicine. According to the Frontier keywords (Figure 3), most research focuses on the antioxidant and liver fibrosis of TMMM, especially after 2009. Chemical metabolites, as the basis of pharmacological research, are another research hotspot.

## 3 Sustainable development of TMMM resources

Chinese herbal resources were initially examined through the Fourth National Survey of Chinese Materia Medica Resources and some challenges were identified. The uneven distribution of TMMM resources and the destruction of natural ecosystems caused by long-term predatory mining make it challenging to access medicinal plant resources. The protection and efficient development and utilization of TMMM resources are therefore critical to the future of TMMM. Since the distribution of herbal resources has obvious geographical characteristics, only by combining herbal resource data with spatial data can we develop a sustainable management model. Resource census, especially when combined with big data, is an effective tool to provide an efficient information transfer system for the survey, recording, and data management of herbal resources. Furthermore, it is necessary to conduct research on the artificial cultivation of Mongolian medicinal plant resources to save endangered species. Effective methods for protecting wild medicinal plant resources, including research on growth suitability, introduction and domestication technology, wild-tending technology, and ecological planting patterns are needed. Finally, the comprehensive use of resources is also an important part of the sustainable use of Mongolian medical resources.

### 3.1 Resource census

**FIGURE 4:**
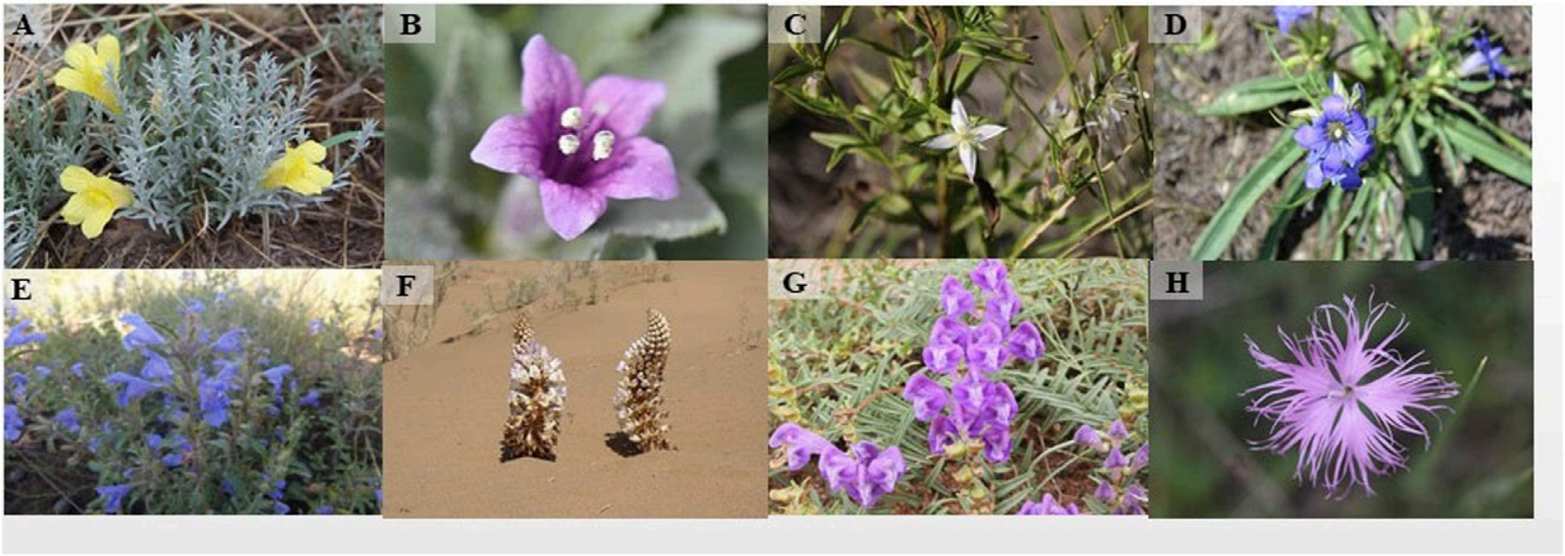
Images of TMMM plants. **(A)** *Cymbaria dahurica* L., **(B)** *Physochlaina physaloides* L. *G. Don*., **(C)** *Lomatogonium carinthiacum* (Wulf.) *Reichb,* **(D)** *Gentiana dahurica Fisch.*, **(E)** *Dracocephalum moldavica* L., **(F)** *Cistanche deserticola* Ma, **(G)** *Scutellaria baicalensis Georgi*, **(H)** *Dianthus superbus* L.

The sustainable use of TMMM resources begins with a clarification of the situation. In ancient times, the discipline involving the use of therapeutic substances in medical remedies was called ‘materia medica’ because it included research on minerals, plants, and animals; most of the materials were plants. There are vast areas in Mongolia and Inner Mongolia, China, with temperate steppe and desert vegetation. The geographical distribution of representative TMMM can be found in Supplementary Figure 1. The complex and varied natural conditions of these areas yield abundant natural and medicinal resources, resulting in traditional Mongolian medicine, with local characteristics influencing the long-term practice used in each region. A total of 2,230 TMMM drug materials are recorded in the *Chinese Materia Medica* (Zheng et al., 2014). These drug materials are sourced from 1,342 species, including 203 plant seeds and fruits, 231 roots and rhizomes, 256 whole grasses, 54 branches and leaves, 83 flowers, 35 barks, 36 rattans, 14 species of fungi and algae, 14 resins, 28 other plant species, 30 species of insects, 260 species of animals and 98 minerals (Nuendagula, 2009; Zhang et al., 2015). These include many well-known medicinal plants, such as *Glycyrrhiza uralensis* Fisch., *Ephedra sinica* Stapf, *Cistanche deserticola* Ma, *Sophora alopecuroides* L. and *Lithospermum erythrorhizon* Sieb. et Zucc., which are native plants in the western region of Inner Mongolia, and *Saposhnikovia divaricata* (Turcz.) Schischk, *Scutellaria baicalensis* Georgi, *Dracocephalum moldavica* L. and *Paeonia lactiflora* Pall from the east (Li, 2019). From 2011 to 2020, the State Administration of Traditional Chinese Medicine organized the Fourth National Survey of Chinese Materia Medica Resources which was established to investigate the medicinal resource system of traditional Chinese medicine and provide effective reference data for its management, protection, and development. Seventy-nine new species were found using 3S technology, namely, remote sensing (RS), geographical information systems (GIS), and global positioning systems (GPS). Of the new species, more than 60% had potential medicinal value (https://www.nature.com/articles/d42473-020-00001-6). Based on the Fourth National Survey of Chinese Materia Medica Resources, 51,180 points data of 2,194 species obtained from the Inner Mongolia census and the 1,044 points data of 687 species supplemented by the Global Biodiversity Information Facility (GBIF) database are explores the diverse areas from different spatial scales and temporal dimensions, which is indicative for the conservation and development of Medicinal plant diversity (Zhang et al., 2022). Moreover, we can not only grasp the species distribution and reserves of Chinese medical resources (including TMMM) in a timely manner but also understand the wild and cultivated trade and demand for TMMM through the resource census. Figure 4 shows some of the medicinal plants found in Inner Mongolia.

### 3.2 Regionalisation research on growth suitability and ecological planting patterns

Ecological and environmental factors such as topography and climate have a major influence on the growth, distribution, and quality of TMMM plant resources. Li et al. (2019) studied 65 wild samples of *Cistanche deserticola* Ma. in Alashan City, Inner Mongolia. They established a quantitative relationship between ecological factors and the quality of medicinal plants using a geographic information system, a maximum entropy model (MaxEnt), and related big data analyses to perform zoning of growth and quality suitability. The most suitable areas for the growth of *C. deserticola* Ma. were southwestern Ejin Banner, Central Alxa Right Banner, and Northern Alxa Left Banner. Dalaihob Town, Ejin Banner, was the best location for the growth of *C. deserticola* Ma. of optimal quality. These findings provide an effective scientific basis for the wild-tending of *C. deserticola* Ma. and site selection for artificial planting bases (Li et al., 2019).

Introduction and domestication technology are practical sciences that utilize the various kinds, forms, purposes, and benefits of organisms. Artificial methods were used to change the environmental conditions for the growth and development of species; this allowed for the introduction and domestication of *Gentiana dahurica* Fisch. in Wuchuan County, Hohhot City, Inner Mongolia. After many years of natural and artificial selection, this species can adapt to the local natural environment and cultivation conditions. The ‘wild to cultivated’ method used for endangered medicinal materials can be applied to other endangered species, thereby reducing damage to wild medicinal plant resources and grassland biodiversity. This method also aids in ensuring the sustainable use of resources. High-quality germplasm should be introduced to avoid problems such as the reduction of species and the biodiversity crisis caused by uncontrolled exploitation of resources (Zhang and Sheng, 2012). Wild-rearing technology is used to cultivate *Lomatogonium carinthiacum* (Wulf.) Reichb in Baotou City, Inner Mongolia and its surrounding areas. This practice supplements areas in which attempts at artificial domestication and cultivation of medicinal plants have been unsuccessful and has helped establish wild cultivation of *L. carinthiacum* (Wulf.) Reichb through artificial breeding, seedling cultivation, irrigation, loosening, and fertilisation. Moderate artificial interventions and various cultivation and management measures have preserved the medicinal characteristics of TMMM plant resources and the genetic diversity of germplasm resources. Although the objective effects of such practices on current growth, environmental benefits, ecological benefits, and other issues must be addressed, this technology promotes the sustainable development of TMMM resources (Li, 2015).

Resource census and artificial cultivation help to create sustainable opportunities for the medicinal plant market. However, quality control of medicinal plants will be necessary before inflow into the market (Hinsley et al., 2019; Bi et al., 2020). The ecological cultivation of medicinal plants is defined as a production mode that uses the ecological niche characteristics of different plant species to efficiently utilize ecological resources between medicinal plants and agricultural and forestry plants (Zhang et al., 2018). Based on the model, the ‘*Astragalus membranaceus* (Fisch.) Bunge-potato-graziery’ ecological planting pattern and its related methods can be used to solve cropping problems that restrict the *A. membranaceus* planting industry in Inner Mongolia. In long-term practice, the repeated, ongoing planting of *A. membranaceus* over multiple years leads to rampant disease and pest infestations; the harvest may even be lost when the incidence is high, causing a sharp decline in the yield and quality of *A. membranaceus*. However, this phenomenon has been avoided by the planting of potatoes at intervals. The aerial part of *A. membranaceus* is used as winter fodder for livestock, and the livestock manure produced by animal husbandry is decomposed and used as organic fertilizer for *A. membranaceus* planting. Promoting such ecological constructs will reduce the use of pesticides, improve the output and quality of *A. membranaceus* and strengthen the sustainable development and utilisation (Zhang et al., 2018). Besides, data on medicinal plant cultivation were acquired for 76 sites from four data stations between 2014 and 2021 in Inner Mongolia, and representative medicinal plants (and their respective organic planting patterns) including *Paeonia lactiflora* Pall., *Saposhnikovia divaricata* (Turcz.) Schischk, *C. deserticola* Ma., *Astragalus mongholicus* Bunge, and *Glycyrrhiza uralensis* Fisch., were selected for analysis. This highlights that the adoption of organic agricultural practices, such as intercropping and imitation of natural conditions, can holistically improve the ecological issues of medicinal plant systems, including sustainability and biodiversity, thereby contributing directly or indirectly to the long-term stability of medicinal plant quality (Jiang et al., 2022).

High-quality medicinal material resources guarantee drug quality and are the basis of TMMM chemistry and pharmacology-related research. The increasing demand for herbal medicines is shifting the focus to finding methods of increasing production while maintaining the high quality of raw materials and promoting the international trade of final products (Zhang et al., 2021). New techniques can also be applied to the study of planting, regionalization, and protection of TMMM resources. The quality of herbal medicines in different regions should be on par with standard regulations in terms of overall structure, suitability of the production area, production environment, and air and soil standards. In particular, researchers are now focused on locating succedaneums, which are endangered botanical drugs. Regionalisation research on growth suitability, introduction and domestication technology, wild-tending technology, and ecological planting patterns are critical for protecting Mongolia’s natural resources.

### 3.3 Comprehensive utilization of resources

Comprehensive utilization of resources is also an important part of the sustainable use of Mongolian medical resources. It can maximize the utilisation efficiency of resources. Although the chemical composition of all parts of plants is the similar, parts other than the useful medicinal parts are discarded, which causes great waste. For example, the naphthoquinones in *Zicao* (*Lithospermum erythrorhizon*), such as shikonin, alkannin, and deoxyshikonin, have been revealed to have a wide range of pharmacological effects, including antitumor, anti-inflammatory, and wound-healing properties (Guo et al., 2019). In other studies, shikonin and its analogues have been reported to have insecticidal activities toward *Culex pipiens* (Michaelakis, et al., 2009), *Tetranychus urticae* (Stefania, et al., 2017), and *Nilaparvata lugens* (Shang et al., 2019). Because these multitarget inhibitors have been shown to be safe for humans in long-term applications, besides the medicinal parts, *L. erythrorhizon* is developed as a plant pesticide, and its integrated use will improve the sustainability of the resource. Although the fruit of *Lycium barbarum* is traditionally used, its stems and leaves have a favorable hypoglycaemic effect (Zhang et al., 2008). However, it is currently only used sparingly. In ginseng, the root is traditionally used, but studies have shown that the active ingredient ginsenosides are more abundant in the above-ground part of ginseng than in the root (Zhao and Wang, 2008).

TMMM development is the material basis of medicine and healthcare career development; it is also concerned with the balance between the natural ecological environment and biological entities. Realization of a peaceful coexistence and harmonious development between man and nature is a valuable objective to be further studied and understood. As the treasure of traditional medicine in China, TMMM has been gradually applied across the country and even the world; it is therefore necessary to ensure the healthy and sustainable development of this resource and to ensure its safe and effective clinical use; it is also necessary to further strengthen the support for the inheritance and innovation of scientific research on Mongolian medicine and practitioner training and to strengthen the protection of TMMM resources.

## 4 Advances in TMMM chemical research

TMMM contains 2,230 species, and 1,342 treatments are generally used (Hai and Song, 2013). The most frequently used and special treatments have been shared from TMMM. A PengYin-SuYe (*Aconitum kusnezoffii*), SenDeng (*Xanthoceras sorbifolia*), ZhuRuHeng-ShaoSha (*Choerospondias axillaris*), and AlaGa-AGaRu (*Syringa pinnatifolia*) have been investigated and compared on a much deeper level (Song, 2012). Studies on the correlation between the chemical metabolites and properties of Mongolian medicine have made significant progress. For example, four kinds of flavonoids found in *Cymbaria mongolica*, tricin-7-*O*-glucopyranoside, Chrysoeriol-7-*O*-glucuronide, apigenin-7-*O*-glucuronide and luteolin, are closely related to antioxidant activity (Dai et al., 2002). Bellidifolin, demethylbellidifolin, swertianolin, and norswertianolin were isolated from *Gentianella acuta*; they have a positive effect on arrhythmia (Lv et al., 2015). Four flavonoids that were isolated from *Gentianopsis barbata*, namely, 1-hydroxide-7-methoxyxanthene (Ping et al., 2003), gentiopicroside (Li et al., 2003), swertiamarin (Wang et al., 2005), and mangiferin (Luo et al., 2012), are effective treatment for hepatitis and acts as a cholagogue. Su et al. isolated 48 metabolites from *S. pinnatifolia*, including lignan, iridoids, phenylpropanoids, flavonoids (Su et al., 2015). Wang et al. isolated 20 metabolites from *Scabiosa tschiliensis*, using spectroscopic methods and structural characterization, including Alcohols, fatty acids, sterols, triterpenoids, flavonoids and 10 stearic acid were isolated from *S. tschiliensis* for the first time, and 18 metabolites were first isolated from *S. pinnatifolia*. The specific 20 metabolites are as follows, flavonoids include luteolin, apigenin, luteolin-7-*O*-glucoside, and apigenin-7-*O*-glucoside; triterpenoids compounds consist of ursolic acid, oleanolic acid, and 2α,3β-Dihydroxyurs-12-en-28-oic acid; phenolic acids metabolites are caffeic acid, chlorogenic acid, and p-Coumaric acid. Other metabolites include β-sitosterol, stigmasterol, daucosterol, palmitic acid, stearic acid, docosanol, tetracosanol, succinic acid, D-Mannitol, and sucrose (Wang et al., 2015). Li et al. identified a compound based on physicochemical properties and spectral data using high performance liquid chromatography (HPLC) and isolated *β*-sitosterol, and for the first time daucosterol and norswertianolin were isolated from *G. acuta* (Li M. et al., 2011).

**FIGURE 5:**
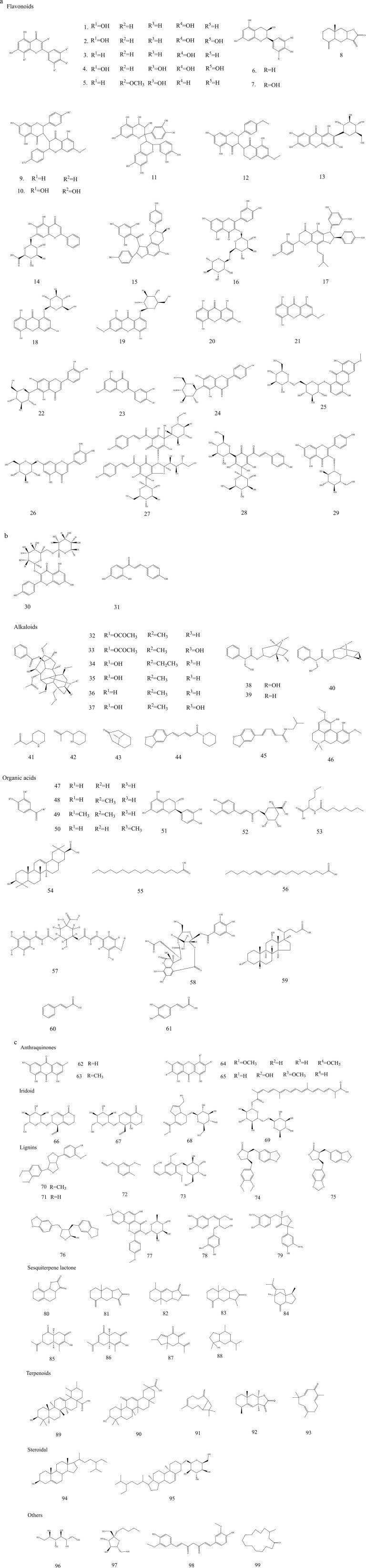
**(a)** Chemical structures of typical TMMM and prescriptions. **(b)** Chemical structures of typical TMMM and prescriptions. **(c)** Chemical structures of typical TMMM and prescriptions.

The chemical metabolites of Mongolian formulae have also been examined. Dong et al. conducted research on effective chemical makeup of Sendeng-4 and isolated anthraquinones including chrysophanol, physcion, and emodin for the first time (Dong et al., 2009; Tian and Dong, 2015). Xin et al. initially isolated eight compounds (kaempferol, daucosterol, p-methoxybenzoic acid, ursolic acid, oleanolic acid, dehydrocostus costunolide, costunolide, and hydrogen) from Shudage-4 (Xin et al., 2016). In recent years, many papers investigating the chemical metabolites of Mongolian medicines have been indexed in SCI. Some of the chemical metabolites in typical Mongolian medicines and prescriptions and their corresponding pharmacological effects are summarised in Table 1 and Figures 5a–c.

**TABLE 1:** Bioactive ingredient and source of typical TMMM and prescription.

*Classification*	Number	Bioactive ingredient	Molecular formula	Source	Pharmacology	Ref.
Alkaloids	1	3-acetylmesaconitine	C_35_H_47_NO_12_	*Aconitum kusnezoffii* Reichb	Anti-inflammatory effects/Analgesia	Zhuo et al. (2007)
	2	3-acetylaconifine	C_35_H_47_NO_13_	*Aconitum kusnezoffii* Reichb	Anti-inflammatory effects/Analgesia	Zhuo et al. (2007)
	3	Aconitine	C_34_H_47_NO_11_	*GaRiDi-5*	Anti-inflammatory effects	Gao et al. (2016)
	4	Anisodamine	C_17_H_23_NO_4_	*Physochlaina physaloides* (L)G.Don	Immunomodulatory effects	Jia et al. (1999)
	5	Beiwutine	C_33_H_45_NO_12_	*GaRiDi-5*	Cardiovascular Protection effects	Gao et al. (2016)
	6	Hypaconitine	C_33_H_45_NO_10_	*GaRiDi-5*	Anti-inflammatory effects/Analgesia	Gao et al. (2016)
	7	Hyoscyamine	C_17_H_23_NO_3_	*Physochlaina physaloides* (L)G.Don	Immunomodulatory effects	Jia et al. (1999)
	8	Isopelletierine	C_8_H_15_NO	*Punica granatum* L	Anthelmintic	Jia et al. (1999)
	9	Mesaconitine	C_33_H_45_NO_11_	*GaRiDi-5*	Anti-inflammatory effects	Gao et al. (2016)
	10	Magnoflorine	C_20_H_24_NO_4_	*Xieri Ga-4*	Anti-inflammatory effects	Ao et al. (2009)
	11	Pelletierine	C_8_H_15_NO	*Punica granatum* L	Anthelmintic	Jia et al. (1999)
	12	Pseudopelletierine	C_9_H_15_NO	*Punica granatum* L	Anthelmintic	Jia et al. (1999)
	13	Piperine	C_17_H_19_NO_3_	*SuGeMuLe-3/XiaoShiWei*	Gastrointestinal Protective Effect	Bao, 2020; Xi et al. (2019)
	14	Piperlonguminine	C_16_H_19_NO_3_	*SuGeMuLe-3/XiaoShiWei*	Cardiovascular Protection effects/Gastrointestinal Protective Effect	Bao and Qi (2011), Xi et al. (2019)
	15	Scopolamine	C_17_H_21_NO_4_	*Physochlaina physaloides* (L)G.Don	Immunomodulatory effects	Jia et al. (1999)
Anthraquinones	16	Bellidifolin	C_14_H_10_O_6_	*Swertia punicea* Helmsl	Cardiovascular Protection effects	Zhang et al. (2013)
	17	Emodin	C_15_H_10_O_5_	*Xieri Ga-4/EW*	Anticancer effects/Inhibit Enlargement of the prostate gland	La et al. (2015)
	18	Methylswertianin	C_15_H_12_O_6_	*Swertia punicea* Helmsl	Cardiovascular Protection effects	Zhang et al. (2013)
	19	Physcion	C_16_H_12_O_5_	*Xieri Ga-4*	Anticancer effects/Inhibit Enlargement of the prostate gland	La et al. (2015)
Flavonoids	20	(−)-epigallocatechin	C_15_H_14_O_7_	*Xanthoceras sorbifolia* Bunge	Inhibition of platelet aggregation	Liang and Zhao (2019)
	21	(2R,3R)-2-(3,4-Dihydroxyphenyl)chroman-3,5,7-triol	C_15_H_14_O_6_	*Xanthoceras sorbifolia* Bunge	Inhibition of platelet aggregation	Liang and Zhao (2019)
	22	7-Methoxylneochaejasmin A	C_31_H_24_O_10_	*Stellera chamaejasme* L	Antibacterial and Antiviral effects	Li et al. (2015)
	23	Apigenin	C_15_H_10_O_5_	*Scabiosa comosa* Fisch. ex Roem. et Schult./*Scabiosa Tschilliensis* Grunning	Inhibition of platelet aggregation	Xin et al. (2016)
	24	Alopecurone F	C_34_H_30_O_9_	*Sophora alopecuroides* L	Inhibitory NO production	Li et al. (2015)
	25	Anhydrosafflor yellow B	C_48_H_52_O_26_	*Carthamus tinctorius*	Cardio-protective effect	Huang et al. (2021a)
	26	Baicalin	C_21_H_18_O_11_	*Xieri Ga-4*	Antibacterial and Antiviral effects	Ao et al. (2009)
	27	Bellidifolin	C_14_H_10_O_6_	*Gentianella acuta*	Antiarrhythmics	Song, 2012; Wang et al. (2017a)
	28	Cynaroside	C_21_H_20_O_11_	*Gentianella acuta*	Antiarrhythmics	Ma et al. (2018)
	29	Dihydrodaphnodorin B	C_30_H_24_O_10_	*Stellera chamaejasme L*	Antibacterial and Antiviral effects	Li et al. (2015)
	30	Desmethylbellidifolin	C_13_H_8_O_6_	*Gentianella acuta*	Antiarrhythmics	Song, 2012; Wang et al. (2017b)
	31	Hydroxysafflor yellow A	C_27_H_32_O_16_	*Carthamus tinctorius*	anti- ischemia/reperfusion	Huang et al. (2021a)
	32	Isorhamnetin	C_16_H_12_O_7_	*Hippophae rhamnoides* L	Anti-inflammatory effects/Antibacterial and Antiviral effects	Dong et al. (2009)
	33	Isoorientin	C_21_H_20_O_11_	*Gentianella acuta*	Antiarrhythmics	Li et al. (2015), Li et al. (2012)
	34	Isoliquiritigenin	C_20_H_18_O_7_	*Glycyrrhiza uralensis Fis*	Cardiovascular Protection effects	Zhan and Yang (2006)
	35	Isovitexin	C_21_H_20_O_10_	*Gentianella acuta*	Antiarrhythmics	Wang et al. (2021a)
	36	Kaempferol-3-*O*-glucoside	C_21_H_20_O_11_	*Carthamus tinctorius*	Cardiovascular Protection effects	Qiburi, Q et al. (2020)
	37	Kaempferol-3-*O*-rutinoside	C_27_H_30_O_15_	*Carthamus tinctorius*	Cardiovascular Protection effects	Qiburi, Q et al. (2020)
	38	Kaempferol	C_15_H_10_O_6_	*Hippophae rhamnoides L./Xieri Ga-4*	Anti-inflammatory effects/Anticancer effects	Zhao and Wang, 2008; Dong et al. (2009)
	39	luteolin	C_15_H_10_O_6_	*Gentianella acuta*	Antiarrhythmics	Li et al. (2015), Li and Zhao (2019)
	40	Mangiferin	C_19_H_18_O_11_	*Sophora pseudochinensis* Hara/*Sophorachirayita* (Roxb. ex Flemi) Karsten	Hepatoprotective effects	Xin et al. (2016)
	41	Myricetin	C_15_H_10_O_8_	*Xanthoceras sorbifolia* Bunge	Inhibition of platelet aggregation	Liang and Zhao (2019)
	42	Norswertianolin	C_19_H_18_O_11_	*Gentianella acuta*	Antiarrhythmics	Song, 2012; Wang et al. (2021a)
	43	Procyanidin B	C_30_H_26_O_13_	*Hippophae rhamnoides* L	Antitumor effects	Dong et al. (2009)
	44	Quercetin	C_15_H_10_O_7_	*Hippophae rhamnoides L./Xieri Ga-4*	Anti-inflammatory effects/Anticancer effects	Zhao and Wang, 2008; Dong et al. (2009)
	45	Ruixianglangdusu B	C_33_H_28_O_10_	*Stellera chamaejasme* L	Immunomodulatory effects	Li et al. (2015)
	46	Rutin	C_27_H_30_O_16_	*Xieri Ga-4*	Anticancer effects	Ao et al. (2009)
	47	Ruixianglangdusu A	C_34_H_30_O_10_	*Stellera chamaejasme* L	Immunomodulatory effects	Li et al. (2015)
	48	Swertianolin	C_20_H_20_O_11_	*Gentianella acuta*	Antiarrhythmics	Song, 2012; Wang et al. (2021a)
	49	Swertiapuniside	C_26_H_30_O_16_	*Gentianella acuta*	Antiarrhythmics	Wang et al. (2021b)
	50	Wogonin	C_16_H_12_O_5_	*Xieri Ga-4*	Anticancer effects	Liang and Zhao (2019)
Iridoid	51	Crocin-3	C_32_H_44_O_14_	*Xieri Ga-4*	Anticancer effects	La et al. (2015)
	52	Gentiopicroside	C_16_H_20_O_9_	*Swertia pseudochinensis* Hara)/*Swertiachirayita* (Roxb. ex Flemi) Karsten	Hepatoprotective effects	Yu et al. (2007)
	53	Geniposide	C_17_H_24_O_10_	*Xieri Ga-4*	Anticancer effects	La et al. (2015)
	54	Swertiamarin	C_16_H_22_O_10_	*Swertia pseudochinensis* Hara/*Swertiachirayita* (Roxb. ex Flemi) Karsten	Hepatoprotective effects	Yu et al. (2007)
Lignins	55	(+)-Pinoresinol Monomethyl ether	C_21_H_24_O_6_	*Stellera chamaejasme* L	Anti-HIV effect	Li et al. (2015)
	56	(+)-Kusunokinin	C_21_H_22_O_6_	*Stellera chamaejasme* L	Anti-HIV effect	Li et al. (2015)
	57	Acuminatin	C_21_H_24_O_4_	*Syringa pinnatifolia*	antioxidant activity	Shao et al. (2014)
	58	Cubebin	C_20_H_20_O_6_	*Syringa pinnatifolia*	antioxidant activity	Shao et al. (2014)
	59	Eugenol	C_10_H_12_O_2_	Xieri Ga-4	Anticancer effects	La et al. (2015)
	60	Eudesmin	C_22_H_26_O_6_	*Stellera chamaejasme* L	Anti-HIV effect	Li et al. (2015)
	61	Isohinokinin	C_20_H_18_O_6_	*Stellera chamaejasme* L	Anti-HIV effect	Li et al. (2015)
	62	Isopinnatifolin	C_20_H_22_O_5_	*Syringa pinnatifolia*	Anti-oxidant activity	Zhang et al. (2014)
	63	Magnolenin C	C_22_H_26_O_9_	*Stellera chamaejasme* L	Anti-HIV effect	(Li et al. (2015)
	64	Pinnatifolin	C_20_H_22_O_5_	*Syringa pinnatifolia*	Anti-oxidant activity	Zhang et al. (2014)
Organic acids	65	(+)-Usnic acid	C_18_H_16_O_7_	*Usnea longissima* Ach	Antitumor effects	Bao and Na (2018)
	66	3,5-di-O-caffeoylquinic acid	C_39_H_48_O_17_	*Gardenia jasminoides Ellis*	Short-term-memory enhancement activities	Yu et al. (2012)
	67	Catechuic acid	C_15_H_14_O_6_	*Clematis aethusifolia* Turcz	Anticancer effects	Bao and Na (2018)
	68	Chlorogenic acid	C_16_H_18_O_9_	*Scabiosa comosa* Fisch. ex Roem. et Schult	Anti-oxidation effect	Xin et al. (2016)
	69	Caffeic acid	C_9_H_8_O_4_	*Syringa pinnatifolia*	Antianginal	Ao et al. (2013)
	70	Deoxycholic acid	C_24_H_40_O_4_	*EW*	Stroke recovery effects	Yutuo (1977)
	71	Ferulic acid	C_10_H_10_O_4_	*Tamarix chinensis Lour/RouDouKou-5*	Cardiovascular Protection effects/Anticancer effects	Guan, 2016; Chang et al. (2016), Ao et al. (2013)
	72	Linoleic acid	C_18_H_32_O_2_	*Xieri Ga-4*	Inhibit Enlargement of the prostate gland	La et al. (2015)
	73	Methyl3,4-dihydroxybenzoate	C_8_H_8_O_4_	*Dianthus superbus* L	Antioxidant effect	Cui et al. (2017a)
	74	Oleanic acid	C_30_H_48_O_3_	*S. pseudochinensis* Hara/*S. chirayita* (Roxb. ex Flemi) Karsten	Hepatoprotective effects	Yu et al. (2007)
	75	Palmitic acid	C_16_H_32_O_2_	*Xieri Ga-4*	Inhibit Enlargement of the prostate gland	La et al. (2015)
	76	Protocatechuic acid	C_7_H_6_O_4_	*Clematis aethusifolia Turcz*	Anticancer effects	Bao and Na (2018)
	77	Terminalic acid	C_20_H_28_O_3_	*EW*	Stroke recovery effects	Yutuo (1977)
	78	*Trans*-Cinnamic acid	C_9_H_8_O_2_	*Syringa pinnatifolia*	Antianginal	Ao et al. (2013)
	79	Veratric acid	C_9_H_10_O_4_	*Xieri Ga-4*	Anticancer effects/Anti-oxidation effect/Anti-inflammatory effects/Antibacterial and Antiviral effects	Ao et al. (2009)
Sesquiterpene lactone	80	1, 13-dihydroxylinolide	C_15_H_22_O_2_	RouDouKou-5	Antibacterial and Antiviral effects	Chang et al. (2016)
	81	14, 15-dinorguai-1, 11-dien-9, 10-dione	C_15_H_20_O_2_	*Syringa pinnatifolia* Hemsl	Antimicrobial activity	Ao et al. (2012)
	82	Alantolactone	C_15_H_20_O_2_	RouDouKou-5	Antiviral effects/Anticancer effects	Chang et al. (2016)
	83	Costunolide	C_15_H_20_O_2_	RouDouKou-5	Gastrointestinal Protective Effect	Chang et al. (2016)
	84	Guai-9-en-4*β*-ol	C_15_H_26_O	*Syringa pinnatifolia* Hemsl	Antimicrobial activity	Ao et al. (2012)
	85	Isoalantolactone	C_15_H_20_O_2_	RouDouKou-5	Antiviral effects/Anticancer effects	Chang et al. (2016)
	86	Isocurcumenol	C_15_H_20_O_2_	XieriGa-4	Anticancer effects	Ao et al. (2009)
	87	Pinnatifone A	C_15_H_20_O_3_	*Syringa pinnatifolia*	Anti-oxidant activity	Zhang et al. (2014)
	88	Pinnatifone B	C_15_H_18_O_3_	*Syringa pinnatifolia*	Anti-oxidant activity	Zhang et al. (2014)
Steroidal	89	β-sitosterol	C_29_H_50_O	*Orobanche pycnostachya* Hance/Xieri Ga-4	Antitumor effects/Anti-oxidation effect/Anticancer effects	La et al. (2015)
	90	Daucosterol	C_35_H_60_O_6_	*Orobanche pycnostachya* Hance	Anti-oxidation effect	Xiao (2017)
Terpenoids	91	Alantolactone	C_15_H_20_O_2_	*EW*		Feng et al. (2015)
	92	Caryophyllene	C_15_H_24_	*Rhododendron micranthum* Turcz	Relieving asthma	Xiao (2017)
	93	Glycyrrhetinic acid	C_30_H_46_O_4_	QingYan LiuWei	Relieving cough and antibechic	Yang et al. (2011)
	94	Ursolic acid	C_30_H_48_O_3_	Xieri Ga-4	Anticancer effects/Inhibit Enlargement of the prostate gland	La et al. (2015)
	95	Zerumbone	C_15_H_22_O	*Syringa pinnatifolia*	Anti-oxidant activity	Feng et al. (2019)
Others	96	Butyl-β-D-fructofuranoside	C_10_H_20_O_6_	*Clematis aethusifolia Turcz*	Cytotoxic effect	Bao and Na (2018)
	97	Curcumin	C_21_H_20_O_6_	*Curcuma Longa L./Xieri Ga-4*	Anti-inflammatory effects/Antitumor effects	La et al. (2015)
	98	D-allitol	C_6_H_14_O_6_	*Orobanche pycnostachya* Hance	Anti-oxidation effect	Xiao (2017)
	99	Muscone	C_16_H_30_O	*GaRiDi-5*	Respiratory effects	Gao et al. (2016)

## 5 Advantages of TMMM in treating modern diseases

**FIGURE 6:**
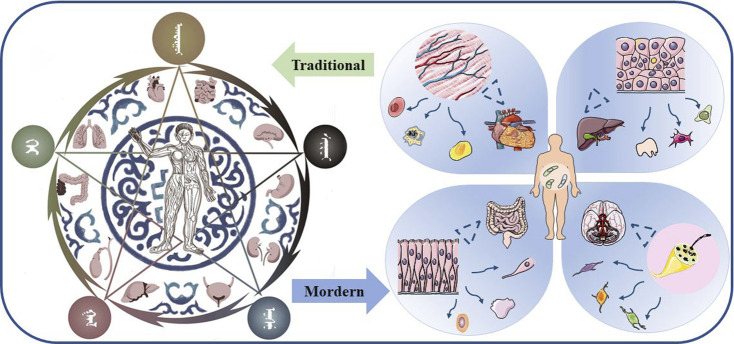
The interconnection and interaction of health between the environment and the body. Traditional Mongolian medicine divides the body organs into five categories and selects different ways to keep healthy according to the changing environment.

Medicinal plants have provided the basis for medical systems throughout human history, and the respective materia medica constitute a large part of these systems. Pharmacological research of Mongolian medicine has made major progress in modern pharmacological interpretation of traditional effects (Zheng et al., 2014). Traditional Mongolian medicine emphasises the unity between human beings and the natural environment, explaining the interconnection and interaction between the activities of organs in the body and the environment (Figure 6) (Kletter et al., 2008). Just like other traditional medicines (Fu et al., 2021), traditional Mongolian medicine regards the behaviour of the human body as a whole during the course of a disease. It defines a healthy individual as having balance within themselves and with their natural environment. Disease is fundamentally related to people’s dietary intake and lifestyle, environmental temperature and other factors, especially chronic diseases (Agula, 2010). For example, a bidirectional relationship exists between type 2 diabetes and climate change; temperature changes can trigger a person’s susceptibility to developing diabetes (Cuschieri and Agius, 2021). A diseased condition represents a deviation from the balance and the role of traditional Mongolian medicine is to restore that balance. Traditional Mongolian medicine uses the principles of nature to distinguish and define different body types or constitutions, each with unique characteristics and responses to the environment, and with predispositions to diseases and reactions to drugs (Agula, 2010). Notably, gut microbiota has been emerging as a new avenue to understanding TMMM, studies have focused on the structure, composition, functionality and metabolites of gut microbiota affected by TMMM so as to conversely understand its theory and mechanisms. Multi-target combination therapy has become an advantage of traditional medicine in the treatment of diseases (Deng et al., 2018; Colombo et al., 2018; Tian et al., 2012). We have chosen to summarise the following characteristic TMMM research topics.

### 5.1 Cardiovascular diseases

Cardiovascular diseases (CVDs) are complicated diseases that encompass a wide range of pathologies, including coronary artery disease and deep vein thrombosis (Lu et al., 2018; Saadeldeen et al., 2020). In allopathy, single target therapy may be an ideal treatment for diseases with clear and simple mechanisms. However, most complex diseases, such as CVDs, cannot be treated through the implementation of a single-target intervention (Kletter et al., 2008). Moreover, there are limitations in the transformation of genotype-based or disease-oriented medicines into personalised and network-based clinical therapies (Emilsson et al., 2008). The treatment approach of traditional Mongolian medicine solves this difficulty. Owing to the influence of long-term diet and lifestyle habits, the hidden dangers of CVDs are more serious (Wang and Xu, 2014). Therefore, Mongolians use unique TMMM to combat CVDs in long-term practice. Traditional Mongolian medicine treats not only the lesions on target organs but also other tissues that are easily affected by the pathological changes. The theories emphasise that every part of the body benefits physiologically and influences a pathology. In TMMM, single botanical drugs, botanical drug pairs, and herbal formulae are composed of diverse and complex metabolites. Their pharmacological effects are generally because of various active metabolites that involve multiple pathways and targets (Lu et al., 2018).

*Syringa pinnatifolia* Hemsl. has been widely used to treat myocardial ischemia induced by excessive Khii (“He-Yi” in Chinese) in the Inner Mongolian regions of China for hundreds of years (Cao et al., 2016; Wang et al., 2016; Xiao, 2017; Yan et al., 2010). These studies demonstrated the effects of Agarwood extracts, major metabolites, and essential oils on myocardial infarction. Recently, Li et al. showed that mice were treated with zerumbone (10, 20, 40 mg kg^-1^·day^-1^) or captopril (50 mg kg^-1^·day^-1^) for 28 days after MI induction, Zerumbone, a humulane sesquiterpene from *S. pinnatifolia*, attenuates cardiac fibrosis by inhibiting the Transforming Growth Factor-beta 1/Sma mothers against decapentaplegic (TGF-β1/Smad) signalling pathway after myocardial infarction in mice (Li et al., 2022). These studies show the process of Mongolian medicine research from demonstrating that TMMMs are effective to finding effective metabolites *in vivo* and *in vitro*. The charm of Mongolian medicine lies in the discovery of new effective metabolites during treatment, which provides direction for the research and development of new drugs. *Fructus Choerospondiatis (‘*GuangZao’ in Chinese)*, which is* derived from the dried fruit of *Choerospondias axillaris* (Roxb.) Burtt et Hill, contains organic acids, phenolic acids, and flavonoids (Figure 4). It has been used extensively to remedy ischemic heart disease and has been shown to have good clinical efficacy (Yang et al., 2016; Shi et al., 2007; Lian et al., 2003; Wu et al., 2014). *Myristica fragrans* (‘RouDouKou’ in Chinese) is rich in fixed and essential oils, triterpenes, and various types of phenolic metabolites and is used to remedy issues related to stomach, heart, and nerve disorders in traditional Mongolian medicine (Abourashed and El-Alfy, 2016; Yutuo, 1977; Kimura et al., 2010; Lee et al., 2012). *F. Choerospondiatis* and *M. fragran* (FCN) have similar functions, and the FCN botanical drug combination is commonly used in traditional Mongolian medicine for the treatment of coronary heart disease (CHD). Lu et al. identified 25 candidate metabolites in *F. Choerospondiatis*, mainly including organic acids, phenolic acids, flavonoids, etc., 38 metabolites in *M. fragran*, including volatile oils, terpenoids, phenols, etc. Network pharmacological analysis predicted that these metabolites could bind to 89 coronary heart disease (CHD)-related targets, such as MAPK14/p38, TP53/p53, F2, etc. These targets are involved in the networks of stress responses, cell adhesion and connections, angiogenesis, and other key CHD-related biological processes (Lu et al., 2018). Further experiments demonstrated the therapeutic effects of FCN (650 mg/kg/day) on isoproterenol-induced myocardial ischemia in rats, indicating that the botanical drug pair may regulate stress and inflammatory responses and inhibit cardiomyocyte apoptosis. Treatment with *M. fragran* or *F. Choerospondiatis* resulted in the downregulation of p38, p53, tumor necrosis factor alpha (TNF-α), interleukin one beta (IL-1β), and Bax to different degrees compared with the model group. FCN also shows a significant therapeutic effect in TMMM formulae such as the *Roukouwuwei* pill, *Guanxinqiwei* preparation, and *Shunqibuxinshiyiwei* pill (Zhao et al., 2012), Feng et al. (2015) administered *Roukouwuwei Pill* at 3g twice daily (6 g·day^-1^) with atorvastatin (20 mg·day^-1^) as a positive control for 4 weeks in unstable angina patients, measuring resistin and hs-CRP levels. Similarly, Kang (2016) used *Guanxin Qiwei Tablet* (3 tablets, 3 times daily; total 2.7g·day^-1^) for 4 weeks in ischemic heart disease, though without explicit positive controls. In animal models, Zhao et al. (2012) treated post-myocardial infarction rats with *Shunqi Buxin Shiyiwei Pill* (0.5 or 1.0 g kg^-1^·day^-1^) and isosorbide mononitrate (10 mg kg^-1^·day^-1^) for 28 days, assessing VEGF-driven angiogenesis. Collectively, these studies highlight dose-dependent efficacy of Mongolian medicines within 4–28 days, often combined with conventional therapies (Feng et al., 2015; Kang, 2016; Zhao et al., 2012). In addition, *Cymbaria daurica* is used to treat diabetes, psoriasis and fetotoxicity in traditional Mongolian medicine. So far, more than one hundred chemical metabolites have been identified in *C. daurica*. These chemical metabolites are divided into flavonoids, phenylethanoid glycosides, phenolic acid, iridoid, and others. Gong et al. isolated n-butanol and ethyl acetate extracts from *C. daurica,* which yielded 10 metabolites that were identified as catalpol, vanillic acid, ajugol, cistanoside F, echinacoside, arenarioside, verbascoside, isoacteoside, apigenin, and tricin. Preclinical studies of these active metabolites have provided the basis for the traditional applications of *C. daurica* (Gong et al., 2020). For example, catalpol attenuates depression-like behaviour in pathological hyperglycaemic states, and its antidepressant mechanism is attributed to the upregulation of PI3K/AKT/Nrf2/HO-1 signalling pathway by reversing the abnormal phosphorylation of PI3K and Akt, as well as the abnormal levels of Nrf2 protein, HO-1 and superoxide dismutase, glutathione peroxidase, glutathione transferase, and reducing glutathione (Zhao et al., 2012).

Eerdun-wurile (EW) is a well-established traditional Mongolian medicine with a long history of clinical application for the treatment of ischemic CVD (ischemic stroke), nervous system diseases (Yu and Hai, 2016; Qiburi et al., 2020), CVDs (Dong et al., 2011), and osteoarthrosis (Eerdun, 2014). Ultra-performance liquid chromatography coupled with quadrupole time of flight mass spectrometry (UPLC-qTOF-MS) analysis has revealed that, among the bioactive chemicals in EW, there are 21 potentially immunoreactive chemicals, including costunolide, pellitorine, and eremanthin. Qiburi et al. found that the petroleum ether extract (50, 100 and 200 μg mL^-1^) downregulates cytokine expression, including the expression of Interferon gamma-induced protein 10 (IP-10), tumor necrosis factor-alpha (TNF-α) and interleukin-1 beta (IL-1β) (Qiburi et al., 2020). In addition, the particular fraction of EW that contains the 21 chemicals was found to efficiently downregulate the expression of key cytokines secreted from M1-polarised microglia and induce inflammation in brain lesions after ischemic stroke. The combined effect of that particular fraction of EW may be responsible for balancing microglial polarisation and subsequent recovery. The mechanism behind this comprehensive formula depends on a variety of bioactive chemicals, which often simultaneously regulate multiple targets through multiple cellular pathways. Thus, the identification of all the metabolites in a herbal combination provides a good basis for the treatment of multiple diseases. Many studies have also shown that EW affects atherosclerosis. Dong et al. showed that EW with 0.1, 0.2 and 0.4 g kg^-1^·d^-1^ decreased total serum cholesterol, triglyceride, low-density lipoprotein cholesterol levels, and aortic plaque area in rabbits, the curative effects of the medium and high doses are remarkable (Dong et al., 2011). The results also showed that EW could inhibit the formation and development of atherosclerosis. The mechanism behind this function may be related to a decrease in blood lipid metabolism and adjustment of blood flow velocity. Gaowa et al. treated middle cerebral artery occlusion stroke in rat models with EW (61.7 and 123.4 mg · 100 g^-1^) for 15 days. Thereafter, differentially expressed genes were identified for their functions during the recovery of ischemic stroke. Microglia markersCluster of Differentiation 68 (CD68), Allograft Inflammatory Factor 1 (Aif1), Colony-Stimulating Factor 1 Receptor (Csf1r) were significantly upregulated upon treatment of the rat model with EW, suggesting that treatment with EW may induce the proliferation, activation, and accumulation of microglia at the stroke site (Gaowa et al., 2018). It is possible that the combined activity of metabolites in individual plant metabolites leads to the clinical effect of EW. A total of 84 hospitalized patients with cerebral infarction were randomly divided into control (n = 40) and the treatment groups (n = 44). In this randomized controlled trial, the treatment group took EW in addition to the standard treatment. The high-dose group was administered EW at a dosage of 0.4 g kg^-1^·day^-1^, the medium-dose group at 0.2 g kg^-1^ day, and the low-dose group at 0.1 g kg^-1^·day^-1^. The control group, however, only received the standard treatment. All other conditions were kept identical between the two groups. After 90 days of treatment, relevant indicators such as blood lipids, serum homocysteine, serum uric acid, and serum inflammatory factors were measured. Then, scores were calculated according to the National Institutes of Health Stroke Scale. The results showed that the additional use of EW could effectively reduce the level of the inflammatory response in patients with cerebral infarction. Specifically, the levels of inflammatory factors in the treatment group were significantly lower than those in the control group. Moreover, EW also decreased the levels of serum homocysteine and uric acid in patients with cerebral infarction. These research findings suggest that EW may contribute to reducing the risk of secondary infarction or disease exacerbation (Yue et al., 2019). The emergence of network pharmacology and the application of molecular docking have greatly promoted the development of Mongolian medicine. On the one hand, the application of these new technologies provides ideas for the study of the pharmacological mechanism of TMMM, linking the chemical metabolites of drugs with potential targets, and predicting the pathway of drug action. On the other hand, it is also of great significance to discover the effective active metabolites in many chemical metabolites of TMMM, and even to discover potential new drugs. Network pharmacology suggested that EW can reduce the expression of epidermal growth factor (EGF) mRNA, promote the expression of epileptic apoptosis-regulating factor, enhance the expression of inhibitory factors in the brain, and inhibit the synchronous firing of neurons in the brain through multiple pathways for pain management. Molecular docking showed that luteolin and 3-methylcyclopentadecanone had strong affinity for Monoamine oxidase A (MAOA), Matrix metalloproteinase 9 (MMP9), and Epidermal growth factor receptor (EGFR). Owing to the small number of cases collected and the low rationality of clinical research design, we should pay attention to the analysis of clinical cases while applying new Mongolian medicine technologies and continuously improving the safety and efficacy of Mongolian medicine in clinical application (Qiburi et al., 2020).

The ethanol extract of Mongolian medicine *Terminalia chebula* Retz. can significantly alleviate aconitine-induced arrhythmia in rats, manifested by reducing heart rate (HR) and QRS amplitude, and increasing RR interval (RR-I) and PR interval (PRQ). Compared with the model group, the high-dose group (100 μg/mL), low-dose group (10 μg/mL), and positive control group (lidocaine, 100 μg/mL) all showed significant reductions in HR and QRS (P < 0.01, P < 0.001), and significant increases in RR-I and PRQ (P < 0.001, P < 0.05). However, the recovery times differed among the three groups: the high-dose group (23 ± 2 min), low-dose group (30 ± 2 min), and positive control group (40 ± 2 min) (Liang et al., 2017). Primary cells were cultured for 3–4 days and then treated with total alkaloids of Aconitum kusnezoffii processed with Chebulae Fructus decoction or raw Aconitum kusnezoffii at concentrations of 0.5, 0.25, 0.125, and 0.0625 mg/mL for 30 and 60 min. The results showed that the toxicity of total alkaloids of Aconitum kusnezoffii processed with Chebulae Fructus decoction on myocardial cells was significantly lower than that of raw Aconitum kusnezoffii, manifested by higher cell viability, lower lactate dehydrogenase (LDH) leakage rate, and lower apoptosis rate. These findings suggest that processing with Chebulae Fructus decoction can reduce toxicity by decreasing the myocardial toxicity of Aconitum alkaloids (Li et al., 2012).

### 5.2 Hepatic diseases

Although different diseases have common pathological mechanisms, the mechanisms of one disease might not be the same in different people. When similar pathological changes (e.g., hepatic injury) are treated using traditional Mongolian medicine, individualised therapy is still administered according to the patient’s symptoms and constitution. Thus, another aspect that validates the efficacy of herbal medicine is individualised therapy that is identical in different people. The different mechanisms of hepatic injury validate the scientific basis of such individualised treatments. During hepatic injury, hepatocytes, neutrophils, and macrophages generate high levels of reactive oxygen species (O_2_^−^, OH and hydrogen peroxide [H_2_O_2_]), which are thought to increase oxidative stress, cause direct cytotoxicity, or act as intracellular signalling mediators (Halina and Agata, 2014). According to the book records, *Sophora flavescens* (Kushen) has been used for a long time in clinical practice of Mongolian medicine and has good clinical effect (Editorial Board of Chinese Materia MedicaState Administration of Traditional Chinese Medicine, 2004). Compound kushen injection contains over 21 metabolites based on HPLC fractionation of extracts from the roots of *S. flavescens* and *Smilax glabra.* (Cui Y. et al., 2017). Researchers have focused on clinical randomised controlled trials of compound kushen injection from 2004 to 2019. They conducted an analysis based on 1,575 patients and the intervention effect of compound kushen injection was evaluated by differences in liver function Aspartate aminotransferase (AST), Alanine aminotransferase (ALT), Total bilirubin (T-BIL) and chronic fibrosis-related indicators (laminin [LN], hyaluronic acid [HA], type III procollagen [COL3], type IV collagen [COL4]). They also speculated that compound kushen injection could be effective for chronic liver fibrosis and cirrhosis (Yang et al., 2021a). Moreover, CCl_4_-induced HCC models and an MCD diet-induced NASH model established that Human hepatic stellate cells (LX-2) were treated with *Compound Kushen Injection* (CKI) at 0.1, 0.5, and 1.0 mg/mL for 24 h CCl4-induced fibrotic mice received CKI (5 or 10 mL/kg/day, i. p.) for 6 weeks. The positive control group received pirfenidone (50 mg/kg/day, oral) compound kushen injection inhibited HSC activation by stabilising the interaction of Smad7/TGF-βR1 to rebalance Smad2/Smad3 signalling and subsequently decreased extracellular matrix formation (Yang et al., 2021b). In fact, a metabolic data-driven systems pharmacology approach has been applied to decode the effective mechanism of compound kushen injection in treating HCC (Wang C. M. et al., 2021). From clinical application to pharmacological mechanism, it has been demonstrated that compound kushen injection treats liver disease through multiple targets without exception, which is precisely the core principle of Mongolian medicine. On the one hand, the occurrence of any disease involves more than one organ, tissue, or cell, and the entire body will react. This is because the blood connects the entire body. Qingganjiuwei powder (QGJWS), which contains nine types of medicinal metabolites, exerts remarkable clinical effects, and is commonly used for hepatic protection. Ge et al. (2020) reported that QGJWS inhibits liver fibrosis in CCl4-induced liver fibrosis model rats. The authors reported that QGJWS treats liver fibrosis via decreases in serum alanine aminotransferase and aspartate aminotransferase levels, based on the results of their immunohistochemistry, Western blotting, and Reverse transcription polymerase chain reaction (RT-PCR) analyses. In that study, Rats received QGJWS (0.5, 1.0, or 2.0 g/kg/day) or silymarin (100 mg/kg/day) treatment significantly downregulated the expression of alpha smooth-muscle action (α-SMA) and type-1 collagen (COL1) and reduced collagen deposition, possibly because of its ability to inhibit the activation of hepatic stellate cells. QGJWS also inhibited the activation of the mitogen-activated protein kinase (MAPK) pathway, and after 8 weeks of QGJWS treatment, the phosphorylation of p38, JNK, and Erk1/2 was significantly downregulated as indicated by the results of Western blot analysis (Ge et al., 2020). Such a change suggests an increase in the degradation of the extracellular matrix, occurring at least in part via restoration of the balance between matrix metalloproteinases and their tissue inhibitors, which may account for the suppression of the MAPK signalling pathway. *Glycyrrhiza uralen sis* Fisch., a leguminous plant, possesses biological activities such as anti-inflammation, immunomodulation, and liver protection. Sa et al. investigated the ameliorative effects of vinegar-soaked licorice on liver fibrosis induced by carbon tetrachloride (CCl_4_) combined with a high-fat diet in rats. The study found that rats administered low-dose (0.8 g kg^-1^), medium-dose (2.5 g kg^-1^), and high-dose (5 g kg^-1^) vinegar-soaked licorice for nine consecutive weeks exhibited reduced serum levels of alanine aminotransferase (ALT), aspartate aminotransferase (AST), hyaluronic acid (HA), laminin (LN), and procollagen type III peptide (PⅢP), as well as decreased expressions of cytochrome CYP2E1 and transcription factor Nrf2 in liver tissues, with alleviated pathological fibrosis in the liver. The therapeutic effect of the high-dose vinegar-soaked licorice (5 g kg^-1^) was comparable to that of the positive control drug colchicine (1.5 mg kg^-1^) and showed a dose-dependent relationship (Sa et al., 2022). *Carthamus tinctorius* L. has been used in many formulae to treat liver injury, such as Gurigumu-7 (Song et al., 2021) and Honghuaqinggan-13 pills (Bao, 2020). Hydroxysafflor yellow A from the flower of *C. tinctorius* could rescue acetaminophen (APAP)-treated zebrafish from morphological abnormalities, enhanced blood circulation, and mitigated APAP-induced toxicity in liver development in liver-specific Red fluorescent protein (RFP)-expressing transgenic zebrafish (Urbain et al., 2008). The active metabolites of *C. tinctorius* with hepatoprotective effect remain to be discovered; this will lay the foundation for the combination of *C. tinctorius* in prescription and the development of new drugs. Drug-induced liver injury is one of the most challenging areas of liver pathology (Li, 2019). Although the clinical administration of anti-tumour drugs kills cancer cells, most patients also have different degrees of hepatic injury after chemotherapy. Thus, as it is difficult to successfully complete the chemotherapy regimen, liver protection has gradually become a conventional treatment during chemotherapy (Li Z. et al., 2014). A study of 80 patients who underwent chemotherapy showed that the application of Mongolian medicine formulae in patients with hepatic injury is significantly effective (Kaplan and Ng, 2017). The observation group was treated with Mongolian medicine, which they began to take while undergoing chemotherapy for 7–10 days. After the treatment, observation of the patients for liver function damage or adverse reaction showed that the Mongolian medicine had curative effects. An index range <20% was considered stable, whereas an index increase of >20% was invalid. The proportion of patients in the observation group without hepatic injury during chemotherapy was 95.00% (38/40). This value was significantly higher than the 72.50% (29/40) recorded in the control group of patients who were not treated with Mongolian medicine; the difference was statistically significant (*P* < 0.05). Diseases, mainly those of a chronic nature, mental disorders, and stress disorders, require long-term treatment with a combination of multiple drugs. In addition, the carriers and targets associated with TMMM can motivate the rethinking of modern drug development for these conditions (Huang et al., 2021b). Traditional medicine is usually a complete medical system integrating physical, psychological, philosophical, ethical, and spiritual health. Also known as a science of self-healing, it is an old global healing system (Lad, 1987). In contrast to the holistic approach of traditional medicines, the therapeutic practice of allopathic medicine often involves paying attention to the unhealthy areas, focusing on the suppression of undesirable symptoms, and aiming for immediate results.

In Ayurveda, the plants known as rasayana are used as rejuvenating and for improving the overall health of anyone undergoing this treatment. The word rasayana literally means the path that rasa takes (rasa: the primordial tissue or plasma; ayana: path). According to Ayurveda, the qualities of rasa-dhatu influence the health of other dhatus (tissues) of the body, as it is the most primary in function and works as the basic unit. Hence any medicinal plant or formulation that improves the quality of rasa (rasayanas), strengthen or promotes the health of all tissues of the body. Apart from promoting good health, increasing the ability to concentrate, improving memory and mood, an important effect of rasayana therapy is increasing resistance to diseases (Wang K. et al., 2021). The rasayana effect is not a specific pharmacological action, but rather a complex response operating through a comprehensive holistic mechanism of regulation of homeostasis. Other than well-known success stories - artemisinin for malaria, and arsenic trioxide for leukaemia - there seemed to be a lack of scientifically proven remedies.

Yet a bit of probing revealed what a complex story this is. Not only are big efforts underway to modernize traditional medicine in China and Japan, but Western medicine is adopting some aspects of the Eastern point of view too. In particular, modern medical practitioners are coming around to the idea that certain illnesses cannot be reduced to one isolatable, treatable cause (Efferth, T., & Greten, H. J. 2012). Rather, a fall from good health often involves many small, subtle effects that create a system-wide imbalance. But do traditional medicines actually work? Their personalized nature makes randomized controlled trials - the gold standard for testing drugs - extremely difficult (Guo et al., 2007) Rarely are two formulations identical. However, as modern medicine becomes more personalized, using biological and genetic markers, it is inadvertently developing the tools to better test traditional medicines. Although artemisinin and arsenic trioxide are the archetypal examples of successful modern medicines mined from traditional Asian medicine, they do not represent the ideal convergence of the two systems (Li, S., & Zhang, B. 2013). There are unique aspects to traditional Asian medicine that could hold great promise if they are artfully investigated. The goal of science should be to rigorously test each claim and sort the medical wheat from the pseudoscientific chaff.

However, for chronic diseases requiring long-term medication, natural materia medica used in traditional medicine have more advantages in reducing side effects and promoting overall regulation of health. Herbal products are used alone or in combination in different regions and at different dosages, and multi-target effects can be observed regardless of the specific means of administration (Efferth et al., 2015).

### 5.3 Gastrointestinal diseases

TMMM has its unique theory to treat gastrointestinal diseases with medicinal taste and property as its core contents (Editorial Board of “Chinese Materia Medica” of the State Administration of Traditional Chinese Medicine, 2004) and can be considered for the treatment of gastrointestinal diseases without surgery (Cui Y. et al., 2017). *Gentianella acuta* (Michx.) Hulten is often used as an antidiarrheal agent in traditional Mongolian medicine, which has been reported to have the main metabolites of xanthones, iridoids, flavonoids, and small amounts of triterpenes and steroids (Urbain et al., 2008; Lv and AuthorAnonymous, 2009; Li M. H. et al., 2011), suggesting certain therapeutic effects on ulcerative colitis (UC). UC is a complex chronic inflammatory bowel disease characterised by colonic ulcers (Kaplan and Ng, 2017). Ni et al. demonstrated the anti-UC role of *G. acuta* and investigated the potential mechanisms behind its action both *in vivo* and *in vitro*. *In vivo* trinitrobenzenesulfonic acid-induced colitis rats were treated with desmethylbellidifolin from *G. acuta* (10, 20 and 30 μg mL^-1^) for 10 days (Ni et al., 2019). The results showed that the mRNA levels of iNOS, IL-6, TNF-α and COX-2 reduced significantly in a concentration-dependent manner. The pharmacological effects of single botanical drugs or botanical drugs combinations are generally owing to the multiple targets and pathways involved in various active monomers. Currently, combination therapies are employed to achieve enhanced therapeutic effects in patients with critical diseases. The combination of herbal medicines has been regarded as the halfway point between single botanical drugs and formulae. The typical combination of herbal medicines exerts a basic synergistic effect. This will likely provide new insights into complex disease mechanisms and new clues for future pharmaceutical development through the understanding of the molecular basis behind the synergistic effects of these holistic herbal remedies. Importantly, multiple targets do not mean that research related to single chemical metabolites should be ignored in TMMM (Che et al., 2013). Several TMMM studies have highlighted the meaningful active extracts found in herbal formulae and single botanical drugs. However, there is also increasing interest in the study of single chemical metabolites. For example, (Zhao et al., 2018), validated that gut microbiota transformed albiflorin to benzoic acid, which is a key intestinal metabolite that can cross the blood–brain barrier and inhibit brain D-amino acid oxidase, thereby improving brain function and exerting antidepressant effects. In conclusion, the mechanisms underlying the multi-target pharmacological effects of TMMM may involve three pathways: First, single botanical drugs and herbal formulae composed of diverse and complex metabolites can act to regulate different targets. Second, when different active metabolites focus on the same target, they trigger alternations in other pathways and thus regulate other targets. Third, oral administration of TMMM agents can lead to multi-target effects via gut microbiota (Xilin et al., 2025).

### 5.4 Others

Future studies should aim to analyse the active metabolites. The perennial plant *Cistanche deserticola*, named ‘Rou Cong Rong’ in Chinese, is considered a mysterious folk medicine because of some interesting associated historical tales. The most famous of these tales’ states that *C. deserticola* saved Genghis Khan’s army when they crossed the deserts. Modern pharmacological investigations have revealed that *C. deserticola* exhibits a broad spectrum of pharmacological functions, such as improvements in sexual function, increase in life span (anti-aging effects), enhancements in learning/memory ability (i.e., anti-dementia effect), and facilitation of regular bowel movements. Chemical composition is also the basis of pharmacological research in Mongolian medicine (Song et al., 2021). Li et al. found through observing the effects of different doses of the combination of three drugs on acute toxicity in mice that LD50 in the prepared *Aconitum kusnezoffii* was 798.77 mg kg^-1^, with that in the *Chebulae fructus* and prepared *A. kusnezoffii* group being 927.27 mg kg^-1^. LD50 in the *Glycyrrhiza uralensis* and prepared *A. kusnezoffii* group was 801.28 mg kg^-1^, and that in the *Chebulae fructus*, *G. uralensis*, and prepared *Aconitum kus nezoffii* group was 1,080.9 mg kg^-1^. Compared with those in the blank group on the first day after administration,the body weight and food intake of mice in the prepared *A. kusnezoffii* group were significantly reduced, and the body weight and food intake of mice in the compatibility group were obviously reduced (Li et al., 2024). Phytochemical investigations of *Cistanche* were initiated in the 1980s, and thus far, more than 150 metabolites have been purified and structurally identified. One study showed that echinacoside, a characteristic phenylethanoid glycoside compound in *C. deserticola*, provided neuroprotection against Parkinson’s disease (PD) in a PD mouse model and in murine N9 microglia by double-targeting dopaminergic neuronal survival and neuroinflammation. In these models, treatment alleviated the activation of microglia and suppression of the NLRP3/CASP-1/IL-1β inflammasome signalling pathway (Bao, 2020). In addition, total glycosides (TGs) and polysaccharides (PSs) identified from *C. deserticola* have been shown to prevent osteoporosis in senescence-accelerated mice by activating the Wnt/β-catenin signalling pathway. The study demonstrated that TGs and PSs decreased the expression of phosphorylated-β-catenin and receptor activator of nuclear factor-κ B ligand (RANKL) while simultaneously upregulating the expression of osteoprotegerin, bone morphogenetic protein-2 (BMP-2), p-glycogen synthetase kinase-3β (p-GSK-3β, Ser9), and osteocalcin (Urbain et al., 2008). Thus, treating PD with *C. deserticola* may also help prevent other diseases such as osteoporosis. These findings demonstrate the considerable advantages of multi-target therapy, the side effects of which are likely to subside, highlighting the multiple possibilities for drug treatment using this method.

Combining molecular, cellular, and analytical techniques will serve to better elucidate the multi-target effects of TMMM. Given that this requires an analysis of vast amounts of data concerning the multi-component and synergistic effects of herbal medicine (Csermely et al., 2005; Huang and Li, 2010), computer-aided methods will help promote expedient and accurate analysis, which can in turn aid in the discovery of new active metabolites and the mechanisms underlying the efficacy of prescriptions that have been empirically used for thousands of years. Table 2 shows how complex diseases can be categorised in TMMM and the corresponding classification within modern medicine. The representative single botanical drugs, metabolites, and their pharmacological effects in TMMM are shown in Supplementary Figure 3. It contains 92 nodes and 144 edges. The nodes represent the compound prescription, representative compound prescription, representative single botanical drugs, species pharmacological effects, and the composition of compound prescriptions of TMMM, and the connected edges represent their mutual relationship. The size of the node is directly proportional to the connection degree value. The larger the area of the node, the larger the connection degree value, indicating that the network between the node and other nodes is more extensive. Betweenness represents the ratio of the number of shortest paths through the node to the total number of paths through all nodes. The network model analysis found that nodes with high connectivity, tightness, and betweenness may be the effective active metabolites s that are common to the medicinal plants and metabolites of Mongolian medicine, which is of great significance for the discovery of new effective monomer metabolites. Given the functional interdependencies between the molecular metabolites in a human cell, a disease is rarely a consequence of an abnormality in a single gene, but reflects the perturbations of the complex intracellular and intercellular network that links tissue and organ systems.

**TABLE 2:** Pharmacological effects of TMMM.

Pharmacological effects	Medicinal	Source	Biological metabolites	Model	*In vivo/In vitro*	Treatment	Duration	Results	Ref.
Hypoglycemic effects	*Schisandra chinensis* (Turcz.) Baill	*Schisandra chinensis* (Turcz.)Baill./Schisandraceae	Extract of *Schisandra chinensis*	Alloxan-induced mice	*In vivo*	100 mg kg^-1^	8days	Blood glucose↓, glucose tolerance↑	Yuan et al. (2002)
	*Hippophae rhamnoides* L	*Hippophae rhamnoides* L./Elaeagnaceae	Extract of ethyl acetate from *Hippophae rhamnoides* leaves	Type 2 diabetic mice were induced by high-sugar and high-fat feed	*In vivo*	20.0 mg/kg	84 days	Insulin↑, hemoglobin ↑, C-peptide content↑, Glycated hemoglobin ↓	Yang et al. (2021b)
Immunomodulatory effects	*Hippophae rhamnoides* L	*Hippophae rhamnoides* L./Elaeagnaceae	Flavonoids from *Hippophae rhamnoides* L	^60^Coγ radioactive ray- induced tissue injury	*In vivo*	10 and 20 mg kg^-1^	7 days	White blood cells↑, bone marrow proliferation activity↑	Liu et al. (2015)
	*Angelica sinensis* (Oliv.) Diels	*Angelica sinensis* (Oliv.) Diels/Umbelliferae	*Angelica sinensis* poly saccharides	Rat immune colitis model	*In vivo*	250 and 500 mg kg^-1^	28 days	CMDI↓, MPO↓, IL2↓, TNF-α↓, NO↓, TGF-β↑	Li and Liu (2005)
	*Polygonatu kingianum* Coll. Et Hemsl	*Polygonatu kingianum* Coll. Et Hemsl./Liliaceae	Water extract	Rats use long-term overload swimming to establish a model of internal heat of yin deficiency	*In vivo*	2.5 and 10 g kg^-1^	17 weeks	Improve immune function (serum immunoglobulins IgA, IgG, IgM), cyclic nucleotide system (plasma CAMP, CAMP/CGMP)	Wu et al. (2014)
Antitumor effects	*Melia toosendan Sieb.* et Zucc	*toosendan Sieb.* et Zucc./Meliaceae	Toosendanin	Establishment of a mouse model of H22 liver cancer xenograft	*In vivo*	0.173 and 0.690 mg kg^-1^	23–24 days	The number and area of thymic lobules↓, Bcl-2↓, Bax↑, Fas↑	Wang et al. (2010)
	*Eucommia ulmoides* Oliver	*Eucommia ulmoides* Oliver./Eucommiaceae	Total flavonoid	H22 tumor-bearing mouse model	*In vivo*	50, 100, and 200 mg kg^-1^	20–21 days	Tumors inhibitory effect↑, Bcl-2↓, Bax↑	Yuan et al. (2014)
	*Schisandra chinensis* (Turcz.) Baill	*Schisandra chinensis* (Turcz.) Baill./Schisandraceae	Polysaccharide	H22 liver cancer cell line- induced mice liver cancer	*In vivo*	10, 20, and 40 mg kg^-1^	7 days	The cellular and organ immune functions↑, tumors inhibitory effect↑	Gan (2013)
Digestive system effects	*Cynomorium songaricum* Rupr	*Cynomorium songaricum* Rupr./Cynomoriaceae	Polysaccharide	Acute gastric ulcer Wistar rat model	*In vivo*	100, 200, and 400 mg kg^-1^	5 days	Inhibit the formation of gastric ulcers in water-stressed rats	Yang et al. (2011)
	*Angelica sinensis* (Oliv.) Diels	*Angelica sinensis* (Oliv.) Diels/Umbelliferae	Polysaccharide	Lead anemia SD rat model	*In vivo*	15, 30, 60 and 120 mg kg^-1^	28 days	Hb↑, rbc↑, hct↑, alad↑	Tian et al. (2012)
Hepatoprotective effects	*Scabiosa comosa* Fisch. ex Roem. et Schult	*Scabiosa comosa* Fisch. ex Roem. et Schult./Dipsacaceae	Water extract	Wistar rat model of acute liver injury	*In vivo*	10 mL kg^-1^	14 days	ALT↓, AST↓, TBA↓, TNF-α↓, IL-6↓, IL-10↑	Chun et al. (2019)
	*Angelica sinensis* (Oliv.) Diels	*Angelica sinensis* (Oliv.) Diels/Umbelliferae	Polysaccharide	Normal rat	*In vivo*	254, 127 and 63.5 mg kg^-1^	23 days	AST↓, ALT↓, MDA↓, lpo↓, SOD↑, GSH-px↑, Nrf2↓	Wang et al. (2021a)
	*Glycyrrhiza uralen sis* Fisch.	*Glycyrrhiza uralensis* Fisch./Fabaceae	vinegar soaked licorice	liver fibrosis induced by carbon tetrachloride (CCl4) combined with high fat diet in rats	*In vivo*	0.8 gkg^-1^ 2.5 gkg^-1^ 5 gkg^-1^	9 weeks	ALT↓, AST↓, HA↓,LN↓, PⅢP↓, CYP2E1↓, Nrf2↓	Sa et al. (2022)
Anti-inflammatory effects	*Corydalis bungeana* Turcz	*Corydalis bungeana* Turcz./Papaveraceae	Total alkaloids	Intradermal injection of type Ⅱ collagen emulsion in mouse tail roots to establish a mouse arthritis model	*In vivo*	50, 100 and 200 mg kg^-1^	40 days	Anti-CⅡ antibody levels↓, CⅡ-induced delayed auricle↓, TNF-α↓, IL-1↓, PGE2↓	(Li et al., 2014)
	*Trollius chinensis* Bunge	*Trollius chinensis* Bunge/Ranunculaceae	Vitexin	Establishment of a mouse model of acute lung injury	*In vivo*	0.3, 1 and 3 mg kg^-1^	6 h	TNF-α↓, MDA↓, total protein content↓, SOD↑, lung wet/dry weight↓	(Li et al., 2015)
	*Iris lactea* Pall*. var. chinensis* (Fisch.) Koidz	*Iris lactea* Pall*. var. chinensis* (Fisch.) Koidz./Iridaceae	Irisquinone	A model of radiation-induced lung injury in rats was induced by^60^Co γ single 20 Gy right whole lung irradiation induction	*In vivo*	15, 30 and 60 mg kg^-1^·d^-1^	24 weeks	The content of hydroxyproline↓, TNF-α↓, IL-1β↓, TGF-β1↓	Wang et al. (2016)
	*Cymbaria daurica* L	*Cymbaria daurica* L./Scrophulariaceae	70% ethanol extract	Rat eczema induced by 1-chloro-2, 4-dinitrobenzene (DNCB)	*In vivo*	50 mg/mL, 250 mg/mL	23 days	Reduce the levels of TNF-α and IL-2 in serum, and inhibit the mRNA and protein expressions of SHP-1, Vav3 and Grb2	Huang et al. (2023a)
Neuroprotective effects	*Rheum officinale Baill*	*Rheum officinale Baill/*Polygonaceae	Anthraquinone	Middle cerebral artery focal embolism (MCAO) rat model	*In vivo*	7.5, 15 and 30 mg kg^-1^	7 days	The symptoms of neurological deficits↓, the percentage of cerebral infarction↓, SOD↑ MDA↓, NO↓, LD↓, IL-1β↓, TNF-α↓, NOS↓, LDH↓	Yu et al. (2019)
	*Piper longum* L	*Piper longum* L./Piperaceae	Total alkaloids	Brain stereotactic unilateral striatum injection of 6-OHDA to establish rat model of Parkinson’s disease	*In vivo*	50 mg kg·d^-1^	7 weeks	SOD↑, GSH-px↑, CAT↑, NOS↓, MDA↓, NO↓, GSH↓	(Zheng et al., 2014)
	*Rosmarinus officinalis* L	*Rosmarinus officinalis* L./Labiatae	rosmarinic acid	The human retinal vascular endothelial cell model was induced by 25 mmol/L high glucose culture medium	*In vitro*	50 μmol/L	3–7 days	The number of migrating cells, the level of apoptosis, the contents of IL-6, IL-8 and TNF-α, the protein and mRNA levels of VEGF, Caspase-3 and BAX decreased, while the protein and mRNA levels of Bcl-2 increased	Wang et al. (2022)
	*Capsicum annuum* L	*Capsicum annuum* L./Solanaceae	Capsaicin	The mouse ischemia/reperfusion injury model was induced by the middle cerebral artery occlusion (MCAO) method	*In vivo*	Capsaicin 1 mg/kg, AMG517 0.5 mg/kg	3 days	The expression of TRPV1 mRNA increased significantly, IL-10 ↑, TNF-α↓	Peng et al. (2020)
Cardiovascular protection	*Schisandra chinensis (Turcz.) Baill*	*Schisandra chinensis (Turcz.) Baill./*Schisandraceae	Schisandra Compound Extract	The rat model of hyperlipidemia was constructed by feeding with high-fat feed	*In vivo*	100 mg/kg, 200 mg/kg	9 weeks	PPARα↑, LXRα↑, CYP7A1↑, SREBP2↓, HMGCR↓	(Wang et al., 2021)
	*Rheum palmatum* L	*Rheum palmatum* L./Polygonaceae	Rhubarb tannins	The rat brain injury model was established by the Feeney free fall method	*In vivo*	100mg/(kg・24 h)	3 days	SOD↑, AQP4↓, GFAP↓	Zheng et al. (2014)
	*Aconitum kusnezoffii Reichb*	*Aconitum kusnezoffii Reichb./*Ranunculaceae	total alkaloids	Prepare primary cells3-4 days	*In vitro*	0.5, 0.25, 0.125, and 0.0625 mg/mL for 30 and 60 min	7 days	cell survival rate↑ LDH leakage rate↓apoptosis rate↓	Li et al. (2012)
	*Terminalia chebula Retz*	*Terminalia chebula* Retz./Combretaceae	Alcohol extract	Rats with arrhythmia induced by aconitine	*In vivo*	high-dose group (100 μg/mL), low-dose group (10 μg/mL), and positive control group (lidocaine, 100 μg/mL)	2 h	HR↓、QRS↓, RR-I↑、PRQ↑	(Liang et al., 2017)

Bridging the gap Although there are many similarities between the Greek and Chinese concepts of health and medicine, the medical systems that arose in the West and in the East are quite distinct. Most notably, a highly reductionist, detailed view dominates in the West, whereas a more phenomenological, descriptive, and systems-based view holds sway in China. In recent decades, Western systems thinkers have started to combine theories from a variety of disciplines, developing an expanded systems view of medicine. Systems thinking, and in particular systems biology, have been recognized as the scientific bridge between Western medicine and traditional medicine models, including traditional Chinese medicine (TCM) (Wang et al., 2010). Figure 6 illustrates how systems-based theories can bridge Eastern and Western models, as well as connecting ancient and modern ideas. The left forward image shows a dynamic correlation network of interactions between various genes, proteins, and metabolites. This nodal network reflects the particularized understanding of the complexity of biochemical pathways and the dynamic organization of the body that characterize Western biomedical science. The right forward imagery is a drawing of the Taoist Inner Landscape. In keeping with ancient Taoist tradition, the drawing provides a poetic description of the complex relationships among the various organ functions of the body. The background of the figure merges two very well-known, almost archetypical, symbols of systems thinking: the Vitruvian Man (Le proporzioni del corpo umano secondo Vitruvio) and the Taiji (the literal translation of which is “great pole”). The Vitruvian Man is by Leonardo da Vinci, a visionary and pioneer of the evidence-based scientific view of the universe. A man is pictured within a square, which reflects the terrestrial aspect of humanity, and a circle, which represents the spiritual realm. The Taiji (often called the Yin-Yang symbol in the West) represents the Eastern, Taoist tradition of systems thinking. It depicts a dynamic relationship between the two components of a duality that encompasses the known universe. Interestingly, the Taiji, which symbolizes humanity as part of an eternal universe, has all the properties of a fractal.

Western medicine and Chinese medicine developed within the context of different cultures and perspectives of the natural world. The more reductionistic approach of Western biomedical sciences has generated tremendous knowledge of anatomy, physiology, histology, genetics, and biochemistry, while the phenomenological approach of Chinese medicine has produced a more holistic understanding of biology. The two concepts are complementary, and combining them to optimally balance detail and context could generate a highly rewarding step forward for medicine. We believe that Western diagnostics would benefit greatly from the integration of broader knowledge of relationships between symptoms, including consideration of TCM descriptions of syndromes. TCM descriptions offer potential directions for detailed, explanatory biomedical research, bringing us closer to a biopsychosocial model of health in which more and more relationships between diseases, psychology, and behavior are uncovered. Because of the highly interconnected nature of the human interactome, it is difficult to study different diseases at the molecular level completely independent of one another.

## 6 Safety issues

In classical literature, the toxicity of TMMM is classified as acute toxic, middle toxic, toxic, slight toxic, and micro-toxic (Li S. et al., 2014). Toxicity is also influenced by processing, compatibility, and drug introduction, as was observed with *Aconitum kusnezoffii* Reichb. The cause of poisoning may be the low quality of medicinal materials, improper processing of medicinal materials, or excessive dosage (Wang, 2003). The theory of TMMM toxicity plays a guiding role in its safe and rational use in clinical practice. However, two critical issues should be highlighted regarding the safe use of TMMM. First, owing to the presence of cyanogenic metabolites such as rhodiocyanosides and lotaustralin, attention should be paid to the chronic use of TMMM. Second, combinational use of TMMM with conventional drugs can lead to botanical drug-drug interactions that may increase the risk of side effects/toxicity. Whether these side effects are TMMM- or patient-related remains to be further explored.

## 7 Challenges in the development of TMMM

With the guidance of traditional Mongolian medicine theory, researchers can determine how to maximise the implementation of its characteristics, effectively protect TMMM resources, improve the quality of the TMMM, and accelerate the development of the industry and of new Mongolian medicine drugs. This is an important proposition for the modernisation and industrialisation of TMMM (Li, 2019).

### 7.1 Inheritance and sustainability

The shortage of TMMM resources is the main factor that could inhibit the sustainable development of the Mongolian pharmaceutical industry (Xiao, 2017). Prior to 1949, the development of traditional Mongolian medicine was slow, and TMMM was hindered by issues with sample collection and quality control, in addition to the limited availability of information on dosage (Zhang et al., 2015). At this time, TMMM relied entirely on wild resources. However, rapid advancements in medicine in recent decades and an emphasis on returning to natural drug sources have created a vast global market for natural drugs, resulting in massive demand for TMMM resources (Chang and Sun, 2012). Interests, uncontrolled mining, excessive farming, and animal husbandry, combined with industrial pollution and environmental climate change, can rapidly reduce, or even endanger many TMMM wildlife resources. Therefore, the conservation of TMMM resources is urgently required (Buqin, 2015). As previously mentioned, methods for protecting TMMM resources, such as regionalisation research on growth suitability, introduction and domestication, wild-tending technology, and ecological planting patterns, can guarantee the development of TMMM. However, although these measures are important, they are not sufficient. In the future, implementation of projects designed to protect wild TMMM resources should be strengthened, and a characteristic TMMM germplasm resource database that allows for large-scale sharing of data related to characteristic TMMM botanicals should be established. TMMM is an intangible aspect of cultural heritage (Wu, 2015) that should be preserved to ensure that the Mongolian medicinal culture is inherited by future generations, and to prevent the loss of the classic theories of TMMM. Scientific researchers should seize this opportunity and focus on TMMM to promote exchanges between China and the rest of the world.

The protection and development of TMMM resources have profound significance for the innovation of modern medicine. Protection, development, and utilisation are linked and rely on each other. Despite the issues that remain to be addressed, regional policies, scientific and technological forces, industry support, legal protection, and joint societal efforts can promote the protection of biodiversity and the efficient development and sustainable utilisation of TMMM. A modern agricultural industrial system for the TMMM pharmaceutical industry is just around the corner, and TMMM is likely to become an important aspect of health with substantial effects on economic development and social progress.

### 7.2 Effective composition and pharmacology

Hundreds of metabolites may be identified in botanical drugs by applying the proposed mass fragmentation pathways to liquid chromatography with tandem mass spectrometry datasets, causing the toxicity of the botanical drugs to coexist with the active metabolites (Liu et al., 2012). TMMM is a special therapeutic system, which involves the interaction of various botanical drugs; thus, its therapies are plagued by the same problem (Si, 2014). Most of the therapies in TMMM have been administered for many years. In addition, given the enhancement of public awareness of the superiority of natural medicine, TMMM has attracted extensive attention and set off an upsurge of research. However, the effective composition of TMMM resources remains indistinct. For instance, previous pharmacological studies of *Cornus officinalis* Sieb. et Zucc (Huang et al., 2017). focused on its protective function in the liver and kidney and the treatment of diabetes and its complications. *Cornus officinalis* Sieb. et Zucc. has been used to treat various diseases in China, Japan, and Korea for thousands of years. Its pharmacological activity, mechanism of action, and other activities must be explored in depth. Some new research methods, such as those of network pharmacology, can be used to predict the effective composition of the plant and the mechanisms behind its pharmacological activities. Then, the pharmacodynamics and the mechanisms of action of TMMM resources have been elucidated through pharmacological experiments, which provide an experimental and theoretical basis for clinical medication (Huang et al., 2017). Research of the chemical metabolites and pharmacology of TMMM resources should follow the frontiers of research. In addition, introduction of advanced concepts, utilisation of more sophisticated instruments, and integration with other disciplines, such as genomics, proteomics, metabolomics, and molecular biology, are necessary for the exploration of the pharmacodynamics and mechanism of action of TMMM resources. Newer 'omics' technologies and poly-pharmacokinetics will also play an increasing role in bridging the gap between the personalised approach of Chinese medicine theory and modern clinical research methodology.

### 7.3 Preparation and standardization

The TMMM preparation is an important part of traditional Mongolian medicine. There are more than 5,000 preparations of TMMM included in Inner Mongolia’s “Mongolian medicine Preparation Database”, among which there are more than 400 kinds of TMMM preparations commonly used in clinical practice of traditional Mongolian medicine (Li et al., 2019). The TMMM preparation similar to that of traditional Chinese medicine, which also follows the principle of “Monarch, Minister, Assistant, Guide”, and metabolites work together to makes traditional Mongolian medicine showing unique effects (Zhang et al., 2013) Traditionally, TMMM dosage forms can be divided into subsidence types and cathartic type. The subsidence types include decoction, powder, pill, medicinal oil, ash, paste, medicinal liquor and so a total of 10 kinds of dosage forms. The cathartic types contain deobstruent, emetocathartic and other five dosage forms. Although maintaining the original nature of medicinal materials, there are still many disadvantages. The traditional formulation of Mongolian medicine has some problems, such as slow onset, low technical content, rough quality standard, unstable efficacy, poor taste, large dosage, inconvenient application, and not easy to be accepted by people. At present, TMMM dosage form added injection, drop pills, tablets, capsules, paste, spray, suppository, lotion, granules, oral liquid and other more than 10 new dosage forms (Wu et al., 2014). With the guidance of the theory of traditional Mongolian medicine, modern scientific methods and advanced preparation technologies were applied to study the chemical metabolites of TMMM and to develop new dosages form of TMMM. These “reform Mongolian medicine with western preparation technology” achievements are more convenient to use, accurate in dosage and highly recognized in the market, and have achieved significant economic and economic benefits (Su et al., 2015).

Although significant achievements have been made in chemical analysis, it is still a substantial challenge to evaluate the quality of TMMM resources in-depth. At present, many achievements have been made in reforming TMMM preparation technology and dosage forms. The development of new TMMM preparation technology conforms to the standards of Western medicine, a practice which has aroused interest in the development of new TMMM methods. Appropriate preparation technology can effectively improve the efficacy of drugs and reduce their side effects, providing a reference for the development of new TMMM dosage forms (Deng, 2010). However, the commonly used dosage forms on the market, such as powder, pill, and decoction, are still traditional, and account for 90% of the total available dosage forms, whereas new dosage forms such as ointment, granules and oral liquids, account for less than 10%. Furthermore, there is no research and development team worldwide with strong scientific and technological innovation abilities in the field of Mongolian pharmaceutical preparations (Gu et al., 2015). Therefore, the effective application of modern science and technology to the systematic study of TMMM preparations and dosage forms is an urgent problem that needs to be solved using current research and development processes.

The reform of Mongolian pharmaceutical preparations should not always emphasise novelty, peculiarity, and specificity, but should conform to the quality standards of TMMM and the requirements of clinical practice. Standardisation is a prerequisite and important guarantor for the modernisation and internationalisation of TMMM (Muge et al., 2017).^.^Coverage of the standard collection of TMMM is incomplete, and most of the standards of TMMM only have source and basic outline descriptions; the standard inspection items are not perfect, and only a small number of TMMM resources have established content determination (Yi, 2007). This situation severely restricts the production, marketing, and clinical applications of TMMM, especially in terms of the safety of medication (Zhang et al., 2015). Thus, it is difficult to obtain public recognition of TMMM at home and abroad. Improvement of the TMMM standard is essential to ensuring its quality stability from the source and promoting its legal application worldwide. Mongolian medicine has a variety of processing methods according to different properties and treatment requirements. Processing methods can be roughly divided into four categories, such as pretreatment, water preparing, fire preparing, and water-fire preparing (Wang, 2003). Water preparing is the use of water, milk, wine, vinegar, children’s urine, and other liquids as excipients to process medicinal materials. In 2010, Chinese pharmacopoeia collected a large number of Chinese medicine tablets, granules, oral liquid, ointment, capsules, and other dosage forms. Only a few of the Mongolian medicine preparations were included in the Chinese Pharmacopoeia, and ointments, oral liquids, etc., were not included, which also requires an increase in the research and development of modern Mongolian medicine preparations (Li et al., 2014a). Driven by advancements in science and technology, Mongolian medicine is experiencing a modernization paradigm shift. The Inner Mongolia Autonomous Region government has recently invested in the establishment of pivotal Mongolian medicine laboratories to facilitate the standardized production of Mongolian pharmaceuticals. Experts recommend that patients receive integrated therapeutic interventions guided by professional Mongolian medical principles in conjunction with contemporary medical practices. This integration of traditional knowledge and modern scientific methodologies is yielding novel approaches to human health.

### 7.4 Compatibility rules of prescriptions

The compatibility rules of prescriptions are a distinctive feature and a highlight of ethnic medicine. The compatibility of Mongolian prescriptions takes the basic theories of Mongolian medicine as the core, emphasizing the treatment of diseases by regulating the balance of the “three roots” (Heyi, Xila, and Badagan) in the human body (Baoyintu, 2007). Its compatibility principles mainly include the theory of sovereign, minister, assistant, and envoy, as well as the three-factor rule of “taste - effect - digestion - taste”. The two jointly constitute the therapeutic logic basis of Mongolian medicine prescriptions (Bao et al., 2025). The concept of “sovereign, minister, assistant and messenger” refers to the combination of various drugs with different effects based on the disease, forming a tight and effective organic whole. Through the synergy of the primary and secondary drugs, the therapeutic effect is maximized and negative reactions are reduced. The compatibility rule of “taste - effect - digestive taste” is based on the theory of the “basic six flavors” (sweet, sour, salty, bitter, pungent, astringent), “eight medicinal properties” (heavy, greasy, cold, dull, light, rough, hot, sharp) and “four digestive flavors” (sweet, sour, bitter, pungent) in Mongolian medicine. Emphasize the synergistic effects of drugs in the processes of taste, efficacy and *in vivo* metabolism (Ji et al., 2021). In a prescription, this is particularly important. Individual drugs may interfere with each other’s taste, but the digestive flavors can assist each other. Although obstacles are caused by differences in taste, they can still enhance each other’s effects in essence. The research on the compatibility of Mongolian medicine prescriptions is still in its infancy at present. Its modern research mainly focuses on the chemical composition analysis of individual medicines and the metabolic process *in vivo*, but there are many deep-seated problems that need to be solved urgently. First of all, there are significant deficiencies at the level of chemical composition research. Due to the influence of factors such as the origin, planting conditions, harvest season and application site of Mongolian medicinal materials, their chemical metabolites have natural variability. More importantly, simple *in vitro* component analysis cannot accurately reflect the true material basis of the drug efficacy of the prescription *in vivo*. It is difficult to clarify the compatibility rules among drugs only from the perspective of chemical component changes. Secondly, there are systematic errors in the existing research techniques and methods. Take serum drug chemistry as an example. Biological differences such as species, age, gender, and breeding environment of experimental animals, as well as differences in experimental conditions such as blood collection time and sample processing, can all lead to deviations in research results and affect the reliability of the data. The most crucial issue is the disconnection between theory and practice. The compatibility of Mongolian medicine has a unique theoretical system of “taste”, “effect” and “digestive taste”. However, modern research rarely involves the influence of these core theoretical factors on the compatibility of prescriptions, making the experimental results often difficult to be reasonably explained by the traditional theories of Mongolian medicine (Temuer and Agula, 2007). Therefore, how to organically integrate modern analytical techniques with the traditional compatibility theory of Mongolian medicine and establish a research method system that can both clarify the material basis and reflect the theoretical characteristics has become the most urgent problem in the current research on the modernization of Mongolian medicine prescriptions.

## 8 Conclusion

In the process of transitioning from traditional practice to scientific development, Mongolian medicine has made great progress in resources, pharmacology, and preparation production. The inheritance and innovation of TMMM preparation techniques are crucial, alongside the acceleration of research into the modern TMMM system. Consequently, it is imperative to augment research into the efficacy, pharmacological attributes, mechanisms of action, and active chemical metabolites of TMMM. Simultaneously, the establishment of a comprehensive policy framework for the development of the Mongolian medicine industry, coupled with the implementation of a scientific policy-making mechanism and the regulation of production standards and monitoring systems for Mongolian medicine, will facilitate the effective integration and expansion of the Mongolian medicine and pharmaceutical industry chain, thereby fostering significant economic advancement in ethnic minority regions. However, compared with traditional Chinese medicine, Tibetan medicine, and other ethnic medicine, the revitalization of Mongolian medicine is still in a weak position; there is a serious lack of traditional literature collation, new drug development progress is slow, there is a lack of scientific research and development, and there is the challenge of ethnic barriers in the inheritance of Mongolian medicine. Specifically, studies of TMMM are fragmentary and scattered. Most focus only on one or a few aspects; Systematic studies will be the focus and direction of future research efforts. Characteristic TMMM, such as *Cistanche deserticola* Ma. of Orobanchaceae, *Gentiana dahurica* Fisch. and *Gentianella acuta* (Michx.) Hulten of Gentianaceae, are undergoing further research, but it is still not systematic.

In future, the characteristics of the complete Mongolian culture, independent traditional Mongolian medicine theory, unique TMMM processing, and other procedures should be maintained in traditional Mongolian medicine (Kim et al., 2018). The application of a combination of clinical practice, consideration of the patient’s symptoms, modern medical technology, and the unique therapy of traditional Mongolian medicine (Mei et al., 2015) can achieve the best therapeutic effect. There is also a need to develop a unified internationally and domestically recognised technical standard system for TMMM. Meanwhile, the construction of planting bases for TMMM should be strengthened to minimise dependence on wild medicinal materials (Tong et al., 2019; Fu et al., 2020; Zhang and Zhang, 2015). The modernisation of traditional Mongolian medicine will assist in the process of its globalisation and make it a medical resource that may be shared worldwide.
