# Supplementary material for: Exploring traditional mongolian materia medica: the path of progress from tradition to modernity
Source: Front Pharmacol. 2025 Jul 29;16:1554448. doi: 10.3389/fphar.2025.1554448 (PMC12339552; doi:10.3389/fphar.2025.1554448)
Supplement: Supplementary file 1 [file Image2.pdf]

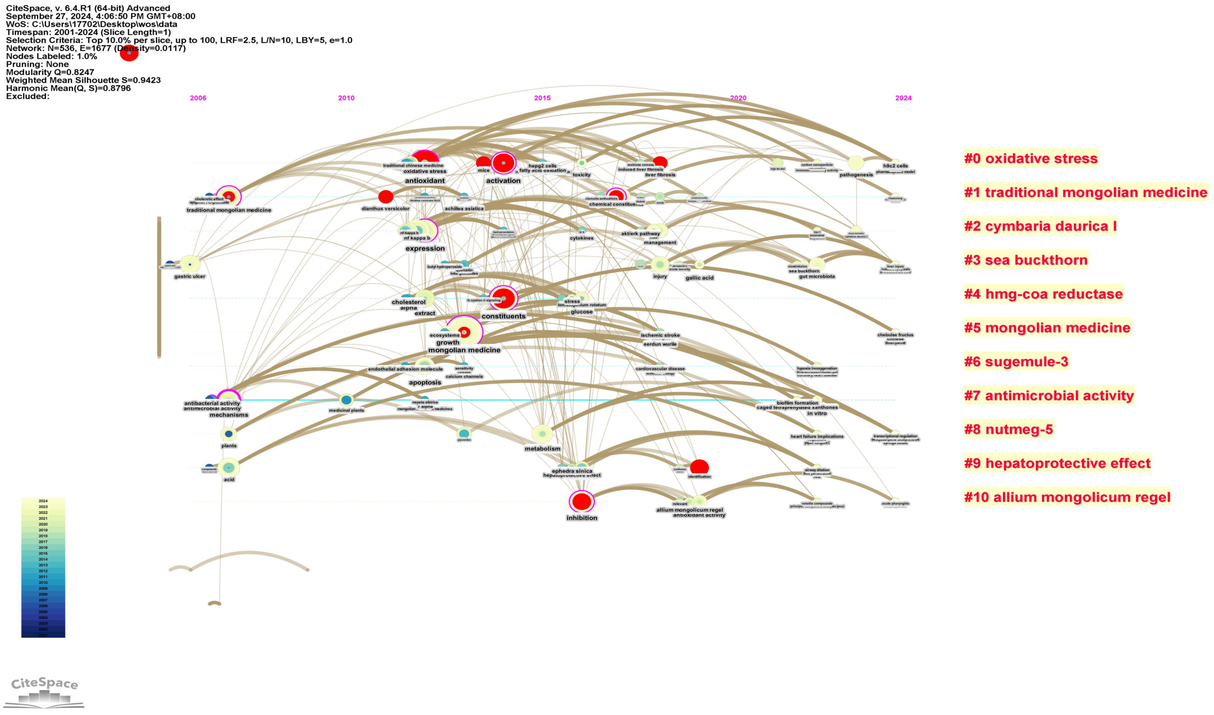

FIGURE 2. First occurrence and development of keywords in each cluster.
