# Supplementary figures and images for: Exploring traditional mongolian materia medica: the path of progress from tradition to modernity
Source: Front Pharmacol. 2025 Jul 29;16:1554448. doi: 10.3389/fphar.2025.1554448 (PMC12339552; doi:10.3389/fphar.2025.1554448)

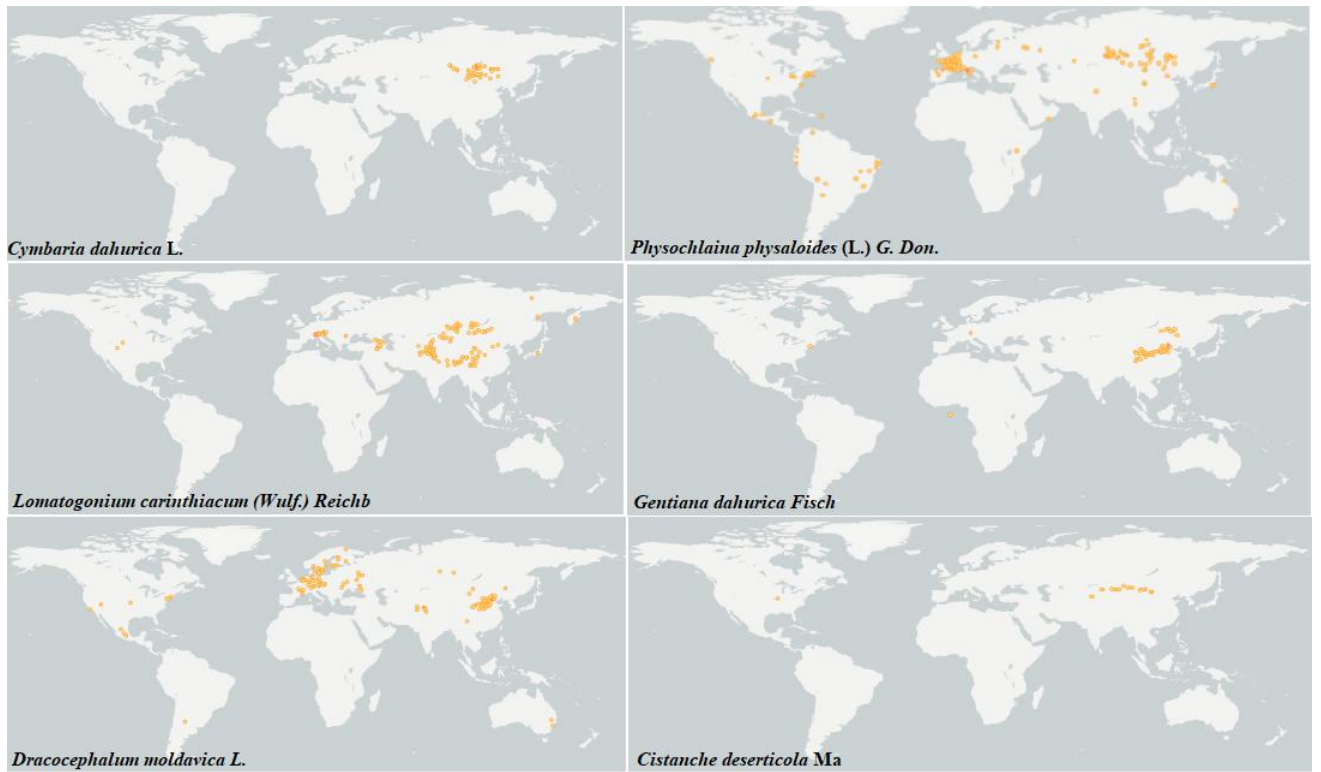

**FIGURE 1.** Geographical distribution of representative TMMM.

Supplement: Supplementary file 3 [file Image1.pdf]
