# Supplementary material for: Exploring traditional mongolian materia medica: the path of progress from tradition to modernity
Source: Front Pharmacol. 2025 Jul 29;16:1554448. doi: 10.3389/fphar.2025.1554448 (PMC12339552; doi:10.3389/fphar.2025.1554448)
Supplement: Supplementary file 2 [file Image3.pdf]

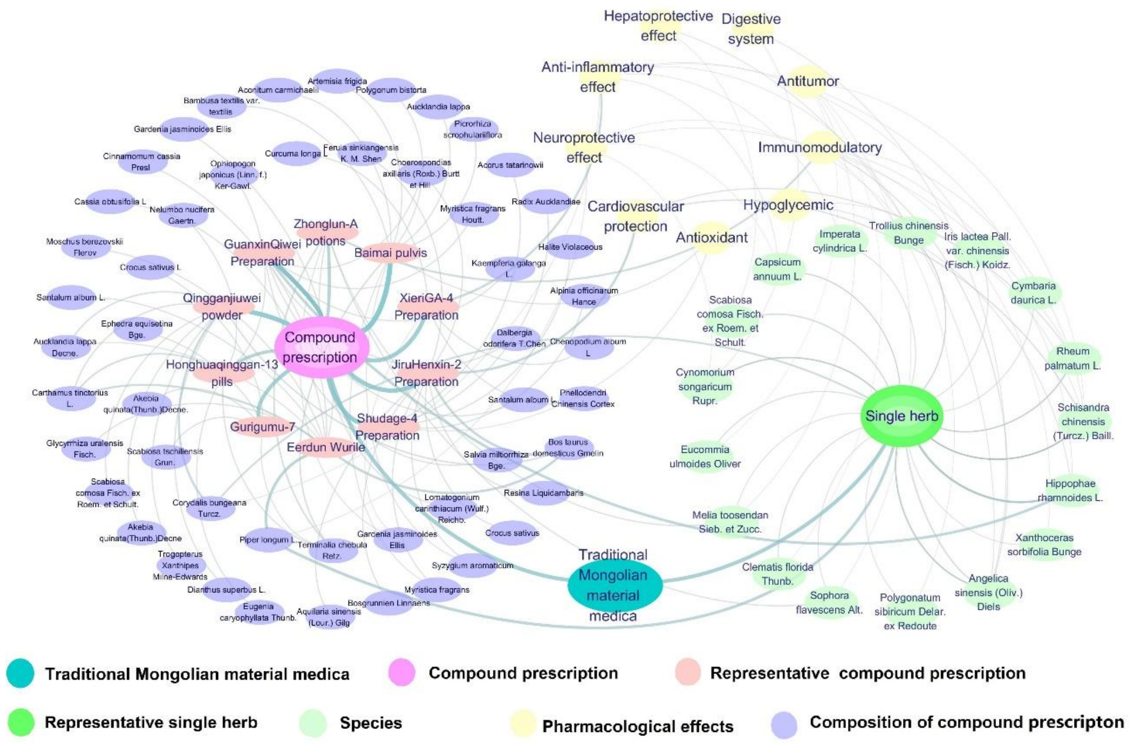

FIGURE 3. **The representative single herbs or compound prescriptions and their pharmacological effects in TMMM.** TMMM can be used alone or in combination to treat the same disease. Purple is the different herbs that make up the formula, associated with a single herb by treating the same disease. Monomer compounds that are truly effective can be found by studying their common active components.
